# Protocols for Subtomogram Averaging of Membrane Proteins in the *Dynamo* Software Package

**DOI:** 10.3389/fmolb.2018.00082

**Published:** 2018-09-04

**Authors:** Paula P. Navarro, Henning Stahlberg, Daniel Castaño-Díez

**Affiliations:** ^1^Center for Cellular Imaging and NanoAnalytics, Biozentrum, University of Basel, Basel, Switzerland; ^2^BioEM Lab, Center for Cellular Imaging and NanoAnalytics, Biozentrum, University of Basel, Basel, Switzerland

**Keywords:** cryo-electron tomography, subtomogram averaging, subboxing, fourier compensation, workflow, viral membrane complex

## Abstract

Cryo-electron tomography allows low-resolution three-dimensional (3D) viewing of cellular organelles and macromolecular complexes present as multiple copies within a tomogram. These structures are computationally extracted and averaged in order to obtain high-resolution 3D structures, and provide a map of their spatial distribution and interaction with their biological microenvironment. To do so, we apply the user-friendly *Dynamo* software package on a tomographic data set. *Dynamo* acts as a modular toolbox adaptable to different biological scenarios, allowing a custom designed pipeline for subtomogram averaging. Here, we use as a textbook example the mitochondrial docking site of the positive-strand RNA Flock house nodavirus (FHV) to describe how *Dynamo* coordinates several basic steps in the subtomogram averaging workflow. Our framework covers specific strategies to deal with additional issues in subtomogram averaging as tomographic data management, 3D surface visualization, automatic assignment of asymmetry and inherent loss of Fourier information in presence of preferential views.

## Introduction

Cryo-electron tomography (cryo-ET) is gaining popularity with structural biologists, because it has the potential to determine the close-to-native *in situ* three-dimensional (3D) structure of frozen-hydrated vitrified biological samples (Lučić et al., 2013; Asano et al., 2016; Oikonomou and Jensen, 2017), ranging from reconstituted membrane proteins (Kühlbrandt, 2015) to eukaryotic lamellas (Villa et al., 2013; Zhang et al., 2016) and tissue (Al-Amoudi et al., 2004; Al-Amoudi and Frangakis, 2013). Vitrification allows rapid freezing of such biological samples preventing ice crystal formation and preserving the physiological structure (Dubochet and McDowall, 1981; Dubochet, 2012).

Electron tomography (ET) requires the acquisition of two-dimensional (2D) projection images of the specimen in a range of orientations (Beck and Baumeister, 2016). To obtain these, the specimen is physically rotated around a single axis inside the electron microscope. This axis is perpendicular to the optical axis of the electron beam. Images are collected at each rotation angle, resulting in a *tilt-series*, i.e., a set of *micrographs*, each one representing a projection of the specimen at a different tilt angle (Tocheva et al., 2010; Gan and Jensen, 2012; Beck and Baumeister, 2016). The tilt-series are then computationally reconstructed into 3D tomograms (Mastronarde and Held, 2017). Unfortunately, the rotation possible, i.e., the tilt range, is physically restricted by microscope geometry and usually only from −60° to +60°. As a result, reconstructed tomograms experience

partial sampling or missing information in Fourier Space (Paavolainen et al., 2014). This missing information exhibits a characteristic wedge-like shape, and is known as the *missing wedge*. In real space, the missing wedge manifests itself as a blurred elongation of the imaged specimen in the direction of the electron beam.

Cryo-ET generally reveals the 3D *in situ* structure of cells, organelles and macromolecular complexes at their nanometer scale, but recently atomic resolution was achieved (Schur et al., 2016). To reach this resolution, a robust image processing method was essential, namely *subtomogram averaging* (STA) (Briggs, 2013). Biological structures of interest that present in multiple copies within a single tomogram, are computationally extracted into subvolumes, also called subtomograms. Subsequently, STA performs a 3D alignment to compute a final average in order to retrieve a 3D structure with enhanced signal. In addition, STA aims to overcome, by computational means, typical limitations linked to cryo-ET, such as the missing wedge, sample geometry (e.g., asymmetry) and low signal-to-noise ratio (SNR) among others (Diebolder et al., 2012; Briggs, 2013; Wan and Briggs, 2016). Software packages specialized for STA aim to address some of this issues (Scheres et al., 2009; Heumann et al., 2011; Huiskonen et al., 2014; Bharat et al., 2015; Galaz-Montoya et al., 2015). This report focuses on a detailed description of the practical steps taken in the *Dynamo* software package to set up a computational STA project for biological tomographic data (Castaño-Díez et al., 2012, 2017; Castaño-Díez, 2017).

*Dynamo* is a software package specialized in image processing for STA that runs in different computing environments (Castaño-Díez et al., 2012). After a brief summary of the main technical details present in *Dynamo*, this report focuses on several functionalities currently included in the software and describes strategies for different scenarios inherent to biological samples such as asymmetry, 3D-orientation and molecular flexibility. Density maps oriented to answer specific biological questions are resolved by following the steps described in this report, along with optimization guidelines and troubleshooting information. Recommended procedures and walkthroughs concerning detailed descriptions of commands and subtomographic analysis are detailed in the online documentation (the *Dynamo* wiki: https://wiki.dynamo.biozentrum.unibas.ch/w/index.php/Main_Page). The practical steps described in this report together with the online documentation encourage users to apply the tools provided by *Dynamo* in their own tomographic data to successfully conduct STA projects. Following the protocol described in this report does not require any particular programming skill. However, a basic familiarity with both MATLAB and system shell scripting in any platform (Mac, Windows or Linux) is of advantage.

## Materials and equipment

### Equipment

- A computer or a computing cluster with at least 1 core and 8 GB of disk space.
- A *Dynamo* installation (version 1.1.319 or latest; available for free: https://wiki.dynamo.biozentrum.unibas.ch/w/index.php/Downloads).
- A MATLAB installation (version R2014b or latest; further details on MATLAB graphical motor when using *Dynamo* are explained in Castaño-Díez et al., 2017).
- A MATLAB installation is recommended but not necessary to use *Dynamo*. A standalone version of *Dynamo* is freely available for all usual platform (Mac, Windows and Linux): https://wiki.dynamo.biozentrum.unibas.ch/w/index.php/Standalone

### Equipment set up

- Data set: Download tomographic data set in the section called “Downloading” here: https://wiki.dynamo.biozentrum.unibas.ch/w/index.php/Advanced_starters_guide or directly at: https://wiki.dynamo.biozentrum.unibas.ch/w/doc/data/fhv/crop.rec.
- Section 9 can be based on the results obtained following the entire protocol described in this report or independently. Data concerning *table*, membrane *model* and *average* for section 9 is available in:
  ◦ *Table*: https://wiki.dynamo.biozentrum.unibas.ch/w/doc/data/fhv/alignmentLocalRefinement.tbl
  ◦ *Model:* https://wiki.dynamo.biozentrum.unibas.ch/w/doc/data/fhv/averagedLocalRefinement.em
  ◦ *Average:* https://wiki.dynamo.biozentrum.unibas.ch/w/doc/data/fhv/membraneModel.omd

## Procedure

The biological tomographic data set used here as a text-book example concerns the mitochondrial docking site of the positive-strand RNA Flock house nodavirus (FHV) (Ertel et al., 2017). This data set is available at https://wiki.dynamo.biozentrum.unibas.ch/w/doc/data/fhv/crop.rec.

FHV forms electron dense spherular invaginations containing RNA fibrils. The spherules attach to the outer mitochondrial membrane (OMM) throughout a necked aperture crowned by a striking cupped ring structure inserted in the OMM, to what we refer as *crown*. This viral macrocomplex (crown) possesses a twelve-fold concentric ring with flanking protrusions referred in this report as *teeth* (Ertel et al., 2017).

### Project design for subtomogram averaging of tomographic data

The Graphical Abstract provides basic information of the workflow followed in *Dynamo* for STA and defines different data generated and used in the software package. In *Dynamo, tables* are the basic metadata system; they are matrices that describe the properties of a set of subtomograms, also referred as particles. A table possesses the metadata of a given data folder that contains cropped particles. As tables are matrices, MATLAB tools are easily customizable to explore and visualize the contents. More information concerning the table and other *Dynamo*'s commands and formats are summarized in Supplementary Table S1 and in the only documentation: https://wiki.dynamo.biozentrum.unibas.ch/w/index.php/Main_Page.

**Graphical Abstract:**
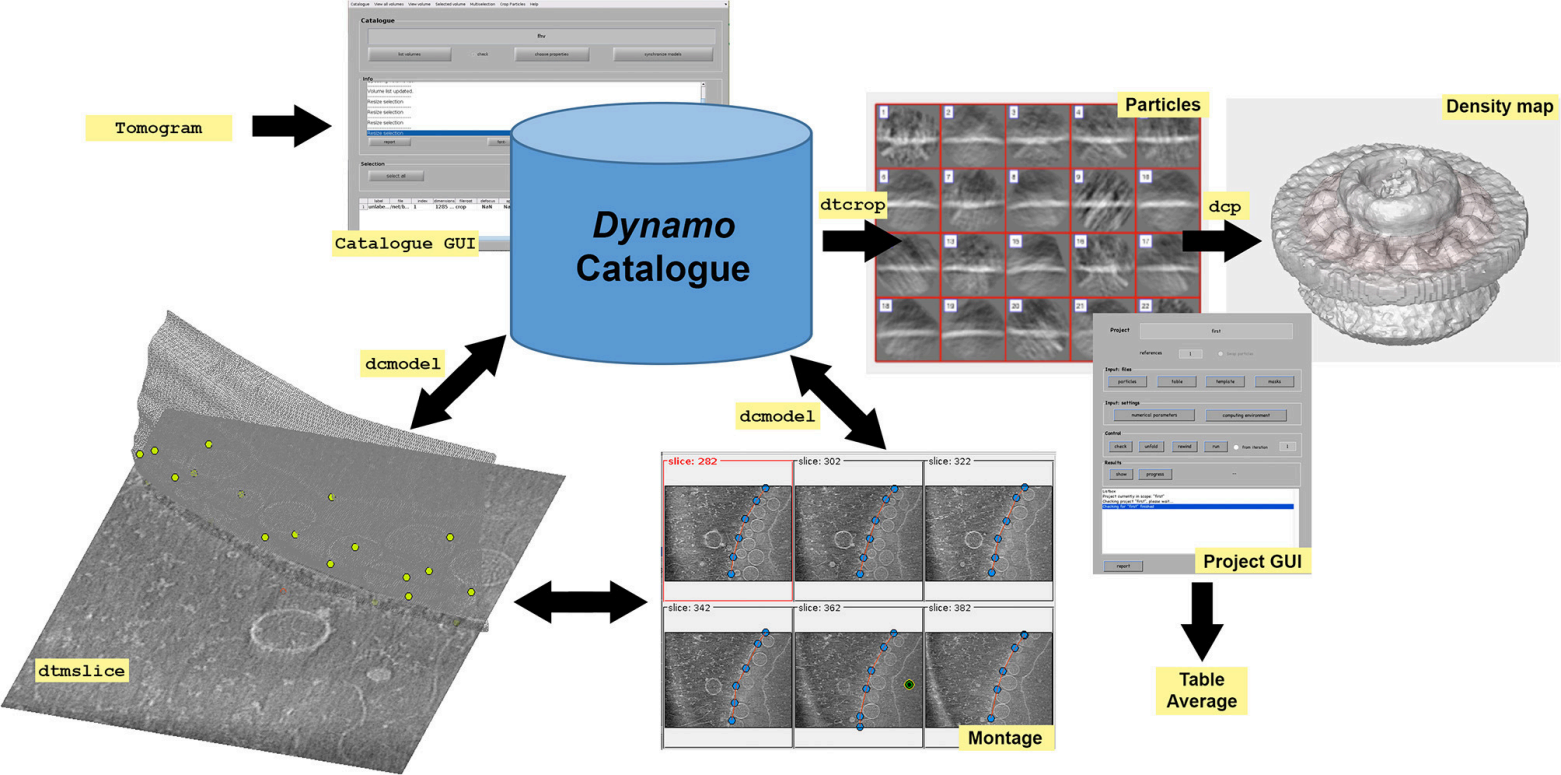
*Dynamo* workflow. Dynamo catalogue* and its GUI (dynamo_catalogue_manager or dcm) provide a robust and simple way to keep track of the transformations and annotations performed on tomographic data sets. The aim is to allow the definition of positions (particles) inside each tomogram, so that subvolumes can be defined and cropped, to produce a unique data folder with its corresponding table, which can then be used to feed a project for alignment or classification. The *catalogue* stores both raw data and annotations performed on the data. In *Dynamo*, annotated tomographic data is defined as geometrical models, therefore *Dynamo* refers to them with the name model. *Dynamo catalogue* keeps track of volumetric processing tasks performed on the tomogram (such as binning or flipping) as well as all manipulations for particle modeling, orientation and geometry. Furthermore, tomogram properties registered in the *catalogue* include paths with the file location, defocus, magnification and a description of the missing wedge among other things. Tomographic data is annotated in dtmslice GUI with the help of *montage* tools that allow the optimal handling and representation of user-positioned points defined in *Dynamo* models, dcmodel (Supplementary Figure S1). Particle positioning can be defined by clicking directly onto the particles or using semi-automation tools integrated in GUIs as dtmslice and *montage* (see Supplementary Figure S1). Particles are extracted from the models using dtcrop to obtain a data folder with the extracted particles and a table that contains the metadata information of the extracted particles. Particles are usually averaged to generate a first template. Then, particles, table and template are used to feed an STA project in the dcp GUI. After running an STA project, an average of the particles and a corresponding table are generated. The density map of the average can be visualized with different tools as indicated in Supplementary Figure S2. *All tools associated with the *catalogue* can easily be used independently at any stage, which means that the creation of a *catalogue* for a tomographic data set is not mandatory. However, we encourage users to organize their tomographic data in a *catalogue* from the beginning. This helps to gain insight into the logic of *Dynamo* and eliminates administrative overheads to localize and track operations in the raw tomographic data.

**Figure 1:**
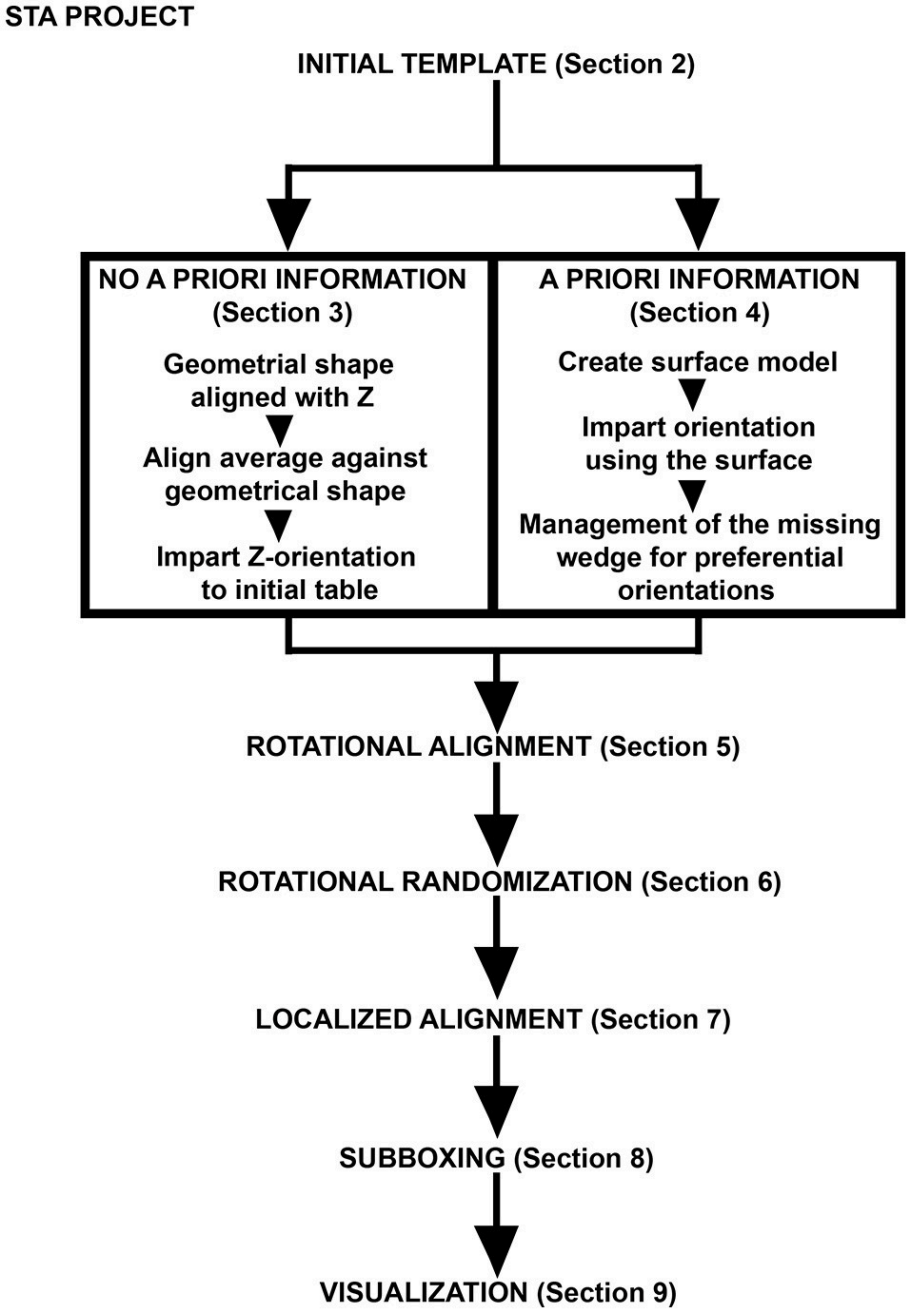
Pipeline for FHV docking site STA. Scheme describing the main steps of the protocol described. The initial template is either oriented by a series of geometrical operations imposed on the particles (no *a priori* information) or by a surface model representing the OMM where the FHV docking sites are inserted (*a priori* information). Management of the missing wedge is an essential step where azimuthal randomization may help, because the particles possess axial symmetry. Different STA projects are designed in order to obtain an optimal density map of the FHV docking site where automatic assignment of asymmetry is managed by subboxing. Eventually, *Dynamo* visualization tools depict the FHV docking sites as anchored macrocomplexes at the OMM in a 3D scene.

The STA strategy followed in this procedure is summarized in Figure 1. Note that for the alignment of the axis of symmetry in the particles the STA strategy divides into two equivalent paths: (1) No *a priori* information and (2) *a priori* information. These two steps focus on aligning the particles along the Z-direction by either applying a set of geometrical tools to the particles or using the OMM to generate a membrane model with geometrical information that is directly applied to the particles inserted in this membrane. Since both paths are equivalent, it is not necessary to perform both of them, unless users wish to improve their practical skills and familiarize themselves with *Dynamo*'s functionalities.

### 1. Tomographic data management

Before starting an image processing procedure, tomographic data should be comprehensively structured. In *Dynamo*, tomograms are organized through *Dynamo catalogue*. In this section, we will create a new catalogue and show how to add, list and bin tomograms. Further, we will explore the tomogram using the *Dynamo*'s tomogram visualization GUI dtmslice, and explain particle picking, extraction and averaging in order to retrieve an initial average.

**1.1** Download the tomographic data set called crop.rec (see Equipment set up). Within the *Dynamo* console, basic metadata of volumetric files can be assessed before loading extensive data sets into memory (a). A quick, lightweight visualization of the tomogram is also available (b).

(a) dfile crop.rec;
(b) dtmshow –otf crop.rec

**INFO:** Header from .rec files is read as regular mrc files. The flag –otf means *on the fly*, and modifies the behavior of dtmshow by not preloading the full tomogram in memory, but to access in disk the individual slices that are needed when inspecting a particular area.

*Dynamo* offers an organized database structured as an archiving system through *Dynamo catalogue* (Castaño-Díez et al., 2017) and its graphical user interface (GUI) dcm, see Graphical Abstract.

**1.2** Create a new *catalogue* named fhv (a), add the crop.rec file (b) to the fhv *catalogue* and ask *Dynamo* to show the content of the *catalogue* concerning the catalogued tomograms (c).

(a) dcm –create fhv
(b) dcm –c fhv –at crop.rec
(c) dcm –c fhv –l tomograms

**INFO:** *catalogue* can be created by the GUI or the command line.

Tomograms are usually processed further before being displayed for modeling and particle picking. *Dynamo* stores in disk the binned version of full-size tomograms to provide a proxy for fast exploration of large volumetric data. In *Dynamo*, binning using a factor of 1 means that 2 pixels become 1 (i.e., a tomogram of size 4 × 4 × 1 k voxels binned in *Dynamo* by a factor of 1 will result in 2 × 2k × 500 voxels tomogram). Loading a full-size tomogram can exhaust RAM resources of the computer and requires a long time to operate the transfer from the disk.

**1.3** Create a binned (factor 1) version of all tomographic data contained in the fhv *catalogue* (a).

(a) dynamo_catalogue_bin(‘fhv’, 1, ‘zchunk’, 300);

**INFO:** ‘zchunk’ refers to the maximum number of Z-slices that are simultaneously contained in memory during the binning process.

Annotated tomographic data is defined as geometrical models, therefore *Dynamo* refers to them with the name model. *Dynamo* models are used to define spatial positions and orientations inside a tomogram where a set of positioned particles are expected to be (see Graphical Abstract and Supplementary Figure S1).

**1.4** Load the binned tomogram into dtmslice (a). In the case of the FHV tomogram crop.rec, we are interested in selecting the locations where the viral spherules are directly interacting with the OMM. Therefore, the neck of the viral spherule is the position where particles are defined by manually clicking on them (Supplementary Figure S1C, green dot, **D**).

(a) dtmslice crop.rec –c fhv –prebinned 1

Pick the particles in the GUI dtmslice as is described in Supplementary Figure S1. Since *Dynamo catalogue* conserves all information from the original tomographic data, even though we are displaying binned data, in dtmslice all distances and coordinates are reported in pixels from the non-binned tomographic data. In this example, the sidelength of the particles is 128 pixels. We recommend ensuring that the particle fits comfortably inside the physical box by leaving enough space, so that the particle sits in the center and away of the edges.

**1.5** Identify the models created so far by using the command dcmodels (a). This command retrieves the number of tomograms containing models and the path to each of the listed models.

(a) dcmodels fhv

**1.6** Extract the table from the model using the method grepTable (a) and print the table in the screen to visualize its content and familiarize with it (b).

(a) dcmodels fhv –i 1 –ws o; m = dread(o.files{1}); t = m.grepTable();
(b) dtinfo(t);

**INFO:** A model read into a workspace variable by dread can be used to extract a table, the standard metadata format in *Dynamo*. Here, we load the output of dcmodels for volume index –i 1 in the workspace variable o. Inside o, there is a field called files (o.files) which contains a cell array of files, each one containing a different model. Then, the first entry (o.files{1}) is read. Finally, we extract the table from the selected model with the function grepTable into the output variable t.

**1.7** Particles are cropped using the dtcrop function (Graphical Abstract). This procedure extracts subvolumes from a defined tomogram using a table and a specific box dimension in pixels (128) for the extracted subvolumes (a)(b).

(a) tomogramFile = m.cvolume.file();
(b) o = dtcrop(tomogramFile,t, ‘particlesData’,128);

**INFO:** At the end of a particle cropping process, *Dynamo* reports information concerning the number of particles cropped, as well as possible excluded particles (cropping box goes beyond the edges of the containing tomogram), destination folder (‘particlesData’ is the name of the folder) and tomographic data source. Furthermore, a table file crop.tbl is generated inside the particle folder (‘particlesData’) with the metadata information of the cropped particles.

**1.8** Average the cropped particles to obtain an initial template and evaluate the data quality for further STA analysis (a). Write the table and the template to disk (b)(c)

(a) oa = daverage(‘particlesData’,'t', 'particlesData/crop.tbl');
(b) dwrite(t,‘raw.tbl’);
(c) dwrite(oa.average,‘rawTemplate.em’);

**INFO:** ‘particlesData’ refers to the name of the data folder containing the cropped particles, ‘t’ is the flag for table and ‘particlesData/crop.tbl' is the path to the table file. The object oa is the output of daverage and contains the resulting geometrical information.

### 2. Project design: particle alignment

A schematic pipeline describing the main steps covered in this report concerning the STA strategy is showcased in Figure 1. *Dynamo* projects can be defined by opening the GUI with the command dcp or directly in the command line. In this example, we explain STA project management using the command line; figures depicting the corresponding procedural steps through the dcp GUI are also shown (Graphical Abstract). In this section we design an alignment project using the particles, the table and the template (average) obtained in the previous section. It is shown how to define appropiate numerical parameters for this project. After running the project an average oriented by the OMM is obtained.

**2.1** Create a *Dynamo* project by the command line:

(a) dcp.new(‘first’,'d','particlesData', 'template','rawTemplate.em', 'masks','default','t', 'particlesData/crop.tbl');

**INFO:** dcp.new creates a new project with the name ‘first’, the particle data ('d') 'particlesData', the template ('template') 'rawTemplate.em', the mask ('masks') as ‘default’ and the table (‘t’) ‘particlesData/crop.tbl’. When typing dcp first, the dcp GUI opens the project called first. In the dcp GUI a button called numerical parameters opens a wizard when clicking on it, with the defined STA project numerical parameters. For numerical parameters definition see Supplementary Table S2.

**2.2** Define the numerical parameters for project first (a), (b):

(a) dcp first
(b) Click on numerical parameters button in the dcp first GUI and introduce the parameter values defined for project first in Supplementary Table S3.

**INFO:** In the project first, the quality of the particles to converge is assessed. The alignment is driven by a common salient feature shared by all picked particles, in this case the OMM where the FHV docking site is inserted. See tip for particle dimensions (Supplementary Table S2). Computational parameters are set specifying the execution mode in CPUs, GPUs or parallelization processing. The environment of the project (MATLAB or a standalone version of *Dynamo*) needs to be indicated (see Supplementary Table S4).

**2.3** Check the project first by either pressing the check button in the GUI or in the command line (a). The same is applied for the buttons unfold (b) and run in the dcp GUI (c) (d):

(a) dvcheck first;
(b) dvunfold first;
(c) first or (d) ./first

**INFO:** The command (c) is used to run the project first when using *Dynamo* within a MATLAB environment. When the standalone version of *Dynamo* is used in a platform (Windows, Linux or Mac) do the same steps described above ((a) and (b)) in the *Dynamo* console; then open a new terminal where *Dynamo* is activated, however the *Dynamo* console must not be started. Run the project first in the terminal where *Dynamo* is activated but not started (d) (see Supplementary Table S4).

**Figure 2:**
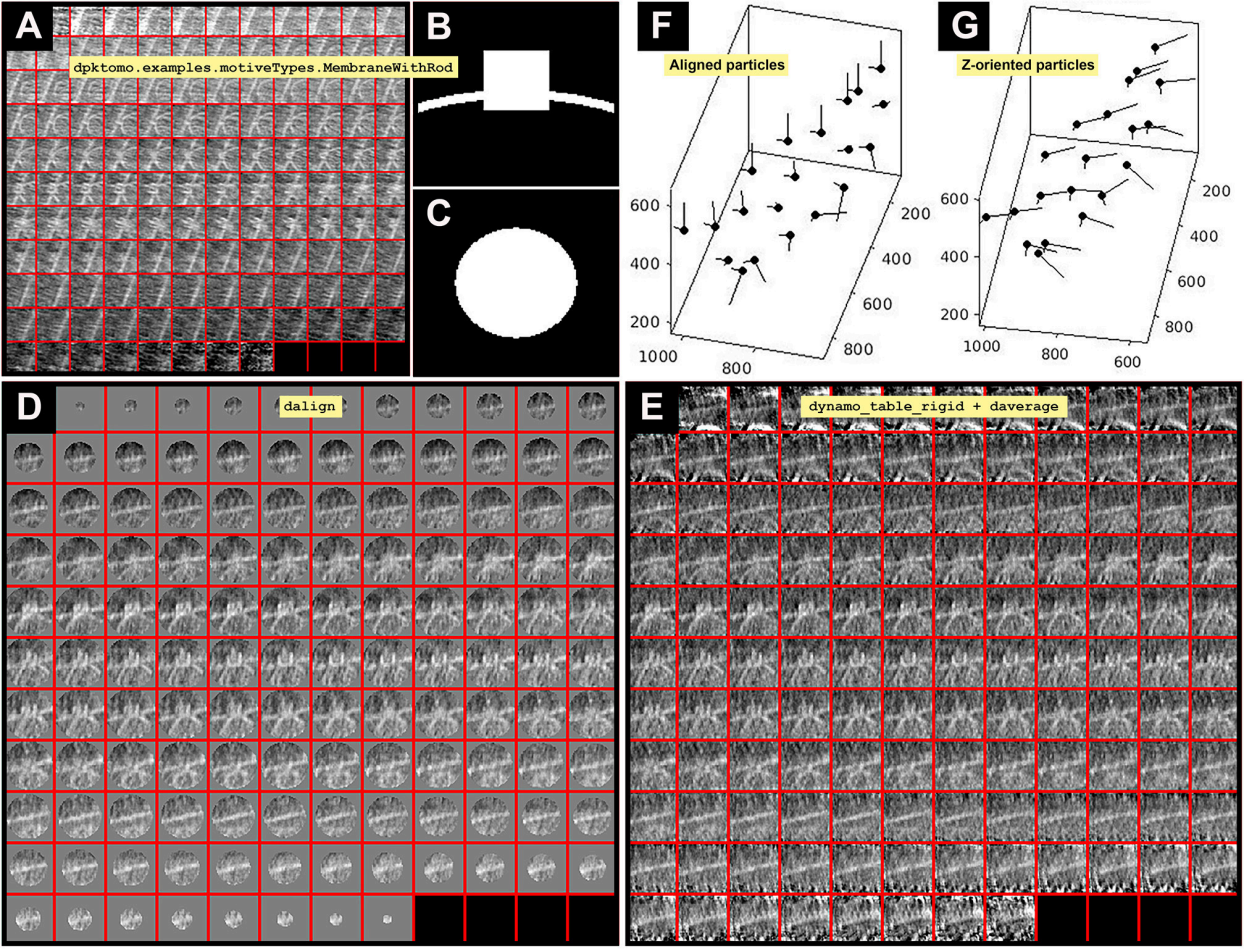
Imparting orientation without geometrical information. **(A)** Slices along Y of the average obtained from project first is defined in the workspace as the variable previousAverage displayed in dview. **(B)** Y view of the mask (slice 64 in dmapview) created by using dpktomo.examples.motiveTypes. MembraneWithRod. **(C)** Z view of the mask (slice 64 in dmapview) created by using dpktomo.examples.motiveTypes.MembraneWithRod. **(D)** Result of applying the mask shown in **(B)** (Y view) and **(C)** (Z view) to the average shown in **(A)** (slices along Y of previousAverage) by using dalign (slices along Y displayed in dview). The output of dalign is contained in the object sal, which encloses geometrical information of the shifts resulting from the dalign command. **(E)** Result of applying the rectified table tr onto the particles shown in **(D)** (slices along Y) by using the dynamo_table_rigid command and averaging the particles (slices along Y displayed in dview). The rectified table tr comes from applying the property inside the object sal (sal.Tp) to the table obtained from project first (tf). **(F)** Graphical representation of the aligned particles (black dots) before applying the rectified table tr. **(G)** Graphical representation of the Z-oriented particles after applying the rectified Z-oriented table tr. The Z orientation computed for each particle is represented by the longest semiaxis.

**2.4** After running the project first, visualize the average obtained (a), and notice that particles are aligned based on the OMM. The STA project first finds the membranes and centers the particles correctly (Figure 2A). Individual particles can be checked (b).

(a) ddb first:a –v
ddbrowse –d first:data –t first:rt

**INFO:** When visualizing data (particles and averages) in ddbrowse the table obtained after running the project first is loaded in order to orientate the particles correctly.

### 3. Aligning the axis of symmetry: no *a priori* geometric information

Once the particles are aligned, their axis of symmetry needs to correspond to the Z-direction. Conceptually, the two first Euler angles jointly represent a reorientation of the main axis of a particle, while the third Euler angle represents a rotation about the new main axis (usually referred to as *azimuthal rotation*). These angles are indicated in the table (particle metadata) at column numbers 24 to 26 with the names tdrot, tilt and narot, respectively (see Supplementary Table S2). They are combined with table columns 4–6 that represent the shifts in X, Y, and Z, respectively, in pixels from the center of the subvolume (Supplementary Table S2). Thus, in this section, we are searching for convergent azimuthal rotations in the particle data set along a possible axis of symmetry that is allocated by convention in the Z-direction. The symmetry axis of the average obtained in section 2 (previousAverage) is reoriented by aligning the average against a Z-oriented template. Therefore, alignment parameters are determined in the table (the Euler angles and shifts) and used to shift and rotate each particle to the Z-direction (Figure 2). Eventually, we will obtain correctly Z-oriented particles used to generate an average for the next STA project. This step is critical to search for convergent azimuthal rotations along the Z-direction in later steps.

Note that this section is equivalent to section 4 below (Figure 1), if section 4 is also performed, make sure to unequivocally name the files concerning table and average from both sections.

**3.1**. To impart the orientation of the axis of symmetry along Z-direction, we first create a template aligned with Z-direction (Figures 2B,C). We use a geometrical shape defined in *Dynamo* as “mask,” however, we will use it as a template in this case. The geometrical shape is contained in the MATLAB workspace object mr (a). Properties of the object mr are defined in (b), (c), (d), (e). Extract (f) and visualize the final mask (g) (Figures 2B,C).

(a) mr = dpktomo.examples.motiveTypes .MembraneWithRod();
(b) mr.sidelength = 128;
(c) mr.rodRadius = 20;
(d) mr.rodShift = [0,0,10];
(e) mr.rodHeight = 10;
(f) mr.getMask();
(g) mr.viewMask();

**INFO:** An advanced tool for creation of geometrical shapes in *Dynamo* is dmask. When invoked without arguments, dmask produces a GUI to create several geometrical shapes (tubes, spheres, cylinders, shells, etc.) where the user can specify the geometrical sidelength of the object and combine different shapes by dynamo_mask_combine.

**3.2** The average obtained by the STA project first (a) is treated as a particle on which we apply a template by using dalign. The template is the volume contained in mr.mask and the average is previousAverage (b). Visualize the resulting Z-aligned average contained in the object sal (c).

(a) ddb first:a -r previousAverage;
(b) sal = dalign(previousAverage,mr.mask, 'cr',360,'cs',30,'ir',0,'dim',32,'limm',1,'lim',[10,10,10]);
(c) dview(sal.aligned_particle);

**INFO:** In (a), the flag –r is used to extract the average obtained in the STA project first into a workspace variable (previousAverage). Note that shift limiting away (‘limm’) is set to 1 (Supplementary Table S2). sal is an object that contains the output of the function dalign.

numerical parameters

('cr','cs','ir','dim','limm','lim') are defined in Supplementary Table S2.

**3.3** Extract the table yield by the project first into the workspace variable tf (a). The object sal contains several properties, one of them is the rigid body transform (a shift and a subsequent rotation) containing the information of the shifts and Euler angles applied to the volume previousAverage to obtain a Z-oriented average. This property is called Tp and can be printed in the screen by typing sal.Tp. Then, sal.Tp is applied onto tf and the result is extracted into the variable tr which is now rotated and shifted according to the rigid body transform indicated in sal.Tp (b).

(a) ddb first:rt -r tf;
(b) tr = dynamo_table_rigid(tf,sal.Tp);

**INFO:** The resulting table, tr, contains the metadata that orients particles in the right Z-direction (upward).

**3.4** Apply the rectified table, tr, to the particles and evaluate the resulting average (a) (Figure 2E). Plot a sketch with the particle orientations obtained from the STA project first before and after applying the rectified table tr (b), (c), (d), (e), (Figures 2F,G).

(a) oz = daverage(‘first:data’, ‘t’, tr, ‘fc’, 1); dview(oz);
(b) figure;dtplot(‘first:rt’,'m','sketch', 'sketch_length',100,'sm',30);
(c) view(-230,30);axis equal;
(d) figure; dtplot(tr,'m','sketch', 'sketch_length',100,'sm',30);
(e) view(-230,30);axis equal;

**INFO:** The normal direction vector of particles in Figure 2G points orthogonal to the OMM whereas in Figure 2F it points in multiple not orthogonal directions to the attached OMM. The flag ‘fc’ means Fourier compensation, in this example set to 1.

### 4. Aligning the axis of symmetry: *a priori* geometric information

**Figure 3:**
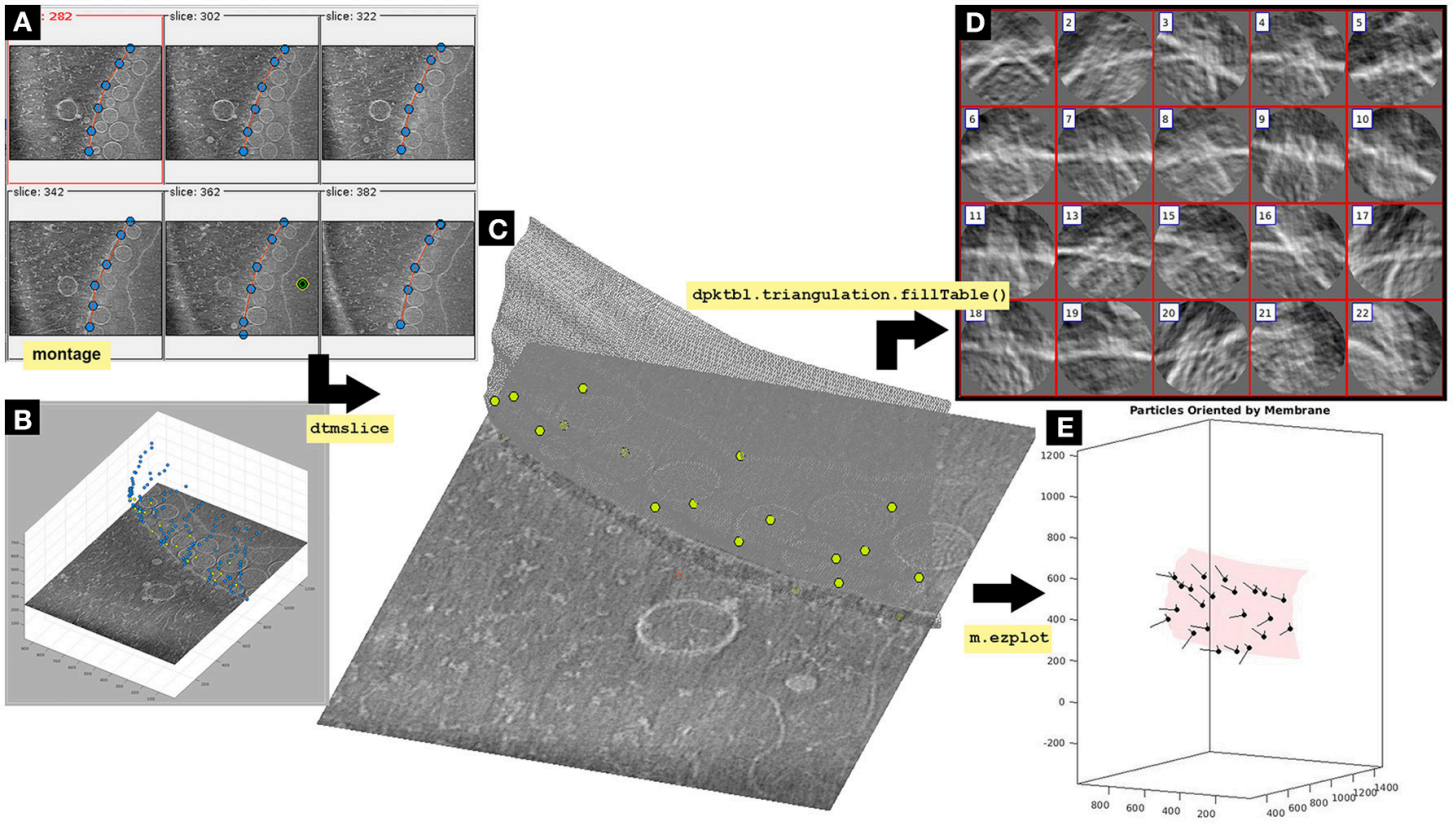
Imparting orientation with a membrane surface *Dynamo* model. The OMM is modeled by a triangulated mesh based on a set of manually picked points. In dynamo tomogram slice (dtmslice) membrane points are picked using a *montage* (*Montage*>*Create Montage of full depicted scene*) to manually click on a set of membrane points. By default, this *montage* shows slices orthogonal to Z, extracted every 20 pixels of the non-binned tomogram **(A)**. *Montage* settings can be modified by the user (*Montage*>*Change montage settings)*. Create a new membrane model in the *montage* GUI following: *Model Pool*>*Create new model in pool (choose type)*>*Surface*. Activate the [c] and [i] *montage* GUI button to insert points on this interface by activating the point switcher in the *montage* toolbar (c-i button). The points are also shown in the 3D model displayed in the depiction box of dtmslice**(B)**. Explore the tomogram along the Z-view in the *montage* GUI by clicking on the arrow buttons at the bottom. The membrane is modeled as a surface where points are manually picked by pressing [i] or left-clicking with the mouse. Importantly, points should be clicked in the same order from slice to slice in order to get a smother triangulation in next steps of the protocol. Points should be picked in the slice where the OMM is clearly seen, and can be deleted by pressing [d] or right-clicking on top of the point that will display an option menu. For semiautomatic detection of points in Z-slices above and below a reference slice, activate the Z-slice where the semiautomatic detection is desired, leave the mouse on top of the activated Z-slice and press the key [o]. The surface that should be modeled is the membrane where the neck of the viral spherules is inserted (Supplementary Figure S1). Importantly, defining the interior side of the membrane model is essential to impart the right orientation to the particles. Leave the mouse on the point where the center is defined and press the keys [shift] and [c]. A green and yellow point is created on the inside of the membrane (**A**, green and yellow dot). The model is saved into the *catalogue* [*Active model*>*save active model into catalogue (disk)* or save all current models in pool by: *Model pool*> *Save all models into catalogue*]. **(A)** *Montage* window representing the XY plane of selected tomogram slices. Blue dots represent membrane surface points linked by a red line that simulates the continuity of the modeled membrane. The green, black and yellow bull's eye point represents the inside side of a mitochondrion. **(B)** Tomogram slice displayed in dtmslice. The OMM is represented as blue dots, this is what the user visualizes in dtmslice when picking the membrane model points in the *montage* GUI. The FHV docking site particles are represented as green dots. **(C)** Tomogram slice displayed in dtmslice. The OMM is represented as a gray triangulated surface containing the FHV docking site particles modeled as green points along the membrane surface. Importantly, the goal of this figure is to visualize the two final models unequivocally [particles (green dots) and membrane]. At this point, no surface triangulation is required, albeit later in this protocol triangulation is performed (see Figure 7). **(D)** Oriented particles obtained by imparting orientation with the membrane using dpktbl.triangulation.fillTable to obtain a table containing the geometrical information of the membrane surface used to impart the correct orientation to the particles. **(E)** Graphical representation of the mitochondrial membrane as a red triangulated surface with the normal vector (black line) of the FHV particles (black dots) pointing in the right direction, toward the outside of the membrane. The Z orientation computed for each particle is represented by the longest semiaxis.

Note: This section is equivalent to section 3 (Figure 1), if section 3 is also performed, make sure to unequivocally identify data concerning table and average from both sections.

The cellular context of an *in situ* biological macrocomplex is preserved in tomographic data (e.g., bacterial secretion systems inserted in the inner membrane of an intact cell). Preserved biological membranes help to assign an initial direction to the particles when applying STA procedures. In the case of the FHV particles, we aim to search for convergent azimuthal rotations along a possible axis of symmetry located by convention along the Z-direction. In this section, we create a geometrical model of the OMM to reorient the particles according to the normal direction of the membrane at their closest point to the particles (Figure 3). Due to preferential orientations imparted by the OMM membrane we experience the effect of the missing wedge in the data set. Here, we show how to attenuate the effect of the missing wedge applying azimuthal randomization to the particles to obtain an accurate average.

**4.1** Display the binned tomogram in dtmlice (a) or (b) when working with the standalone version, and follow the instructions described in Figure 3 to create a surface model of the OMM saved to disk. Display a list of the current models contained in the fhv *catalogue* (c). Translate the model into a workspace variable (c) that is read into memory (d). Use the membrane surface model to impart orientation by extracting the table contained in the model (e). Apply the defined interior side of the membrane surface model to the particles (e). Plot the membrane mySurface (f), press the button surface in the m.ezplot GUI, and add the table containing the normal vector of the particles correctly oriented by the membrane surface model to the plot (g) (Figure 3E).

(a) dtmslice @{fhv}1 -prebin 1;
(b) /dtmslice @{fhv}1 -prebin 1;
(c) dcmodels fhv -nc mySurface -ws output
(d) m = dread(output.files{1});
(e) tOrientedBySurface = dpktbl.triangulation.fillTable (m,'raw.tbl');
(f) tConsistent = dynamo_table_flip_normals (tOrientedBySurface,'center',m.center);
(f) m.ezplot();
(g) dtplot(tConsistent,'m','sketch', 'sketch_length',100,'sm',30);

**INFO:** The membrane surface normal determines the orientation of a particle; the directionality (in- or outwards) is determined by the interior of the membrane surface selected when creating the membrane surface model (Figure 3). mySurface is the name given to the surface model, if no name was given *Dynamo* saves the surface model with the default name mmembraneByLevels. To change the name of the active model, follow in dtmslice GUI: *Active model*>*Change name of active model*.

**4.2** Management of the missing wedge: Particles are aligned and oriented with relation to a membrane, in this case, no particular preference for rotations of the particles about the normal direction has been applied (i.e., azimuthal rotations). Since the particles have a preferential orientation, an average created using these particles has a strong missing wedge.
  Average the particle against the table (a) and visualize the Fourier weight (fweight) (b).

**Figure 4:**
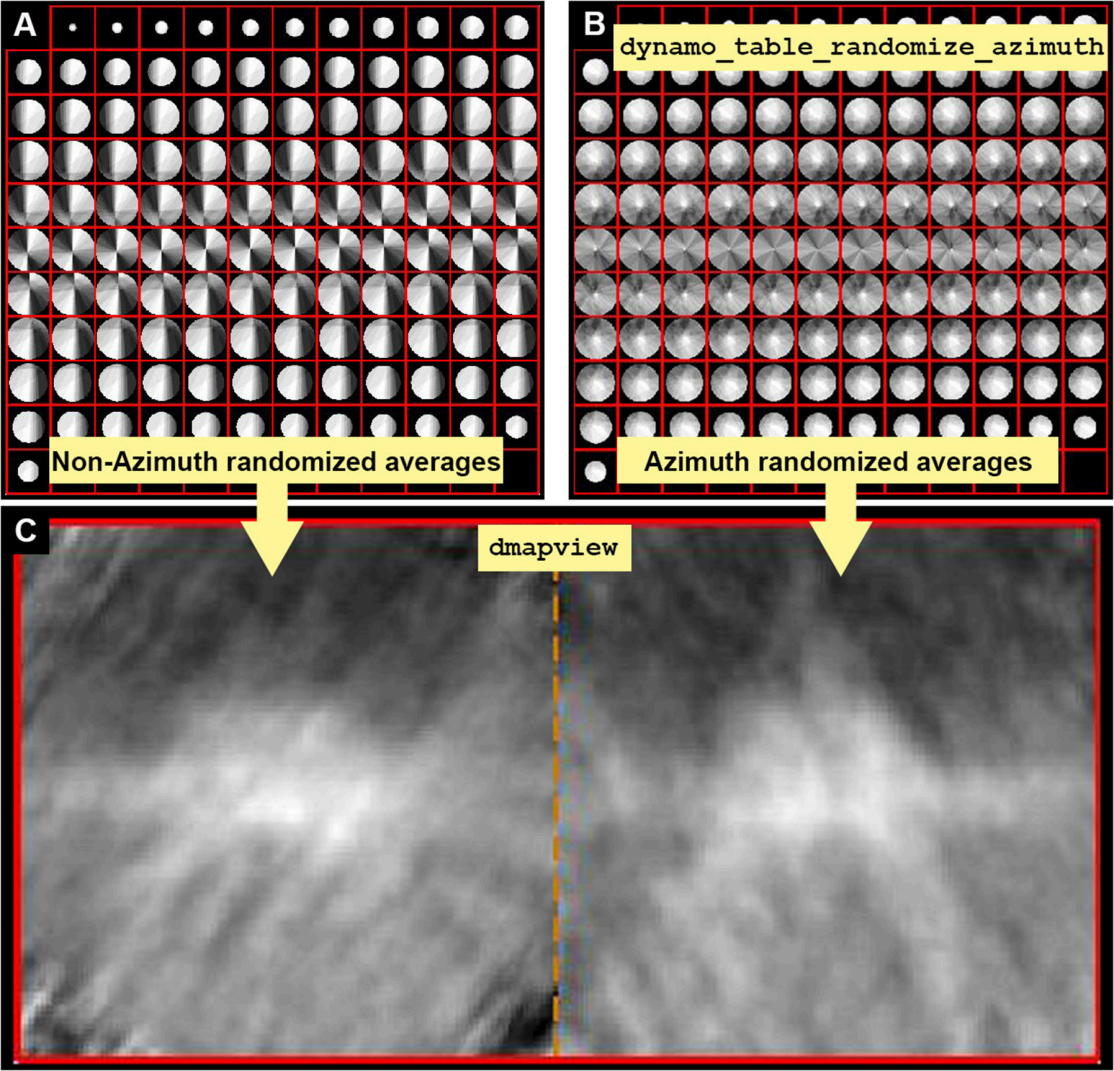
Missing wedge and Fourier compensation. **(A)** Z view of the Fourier components present when no azimuthal randomization is applied. Bright values mean that the Fourier component is present in many particles, indicating preferential orientations. **(B)** Z view of the Fourier components present when applying azimuthal randomization by randomization of the table using the function dynamo_table_randomize_azimuth. **(C)** In dmapview the effect in direct space is shown by depicting both averages, with and without randomization side to side (dmapview({oa.average,oaRandomized.average});). The effect of the missing wedge is seen as similarly oriented stripes in **(C)** (left panel).

Note: This treatment is not suitable when the membrane containing the complexes of interest is orthogonal to the electron beam as it will not contribute to fill the missing wedge.

(a) oa = daverage(‘particlesData’,'t', tConsistent,'fc',1);
(b) dview(oa.fweight);

**INFO:** In a *Dynamo* table the azimuthal angle is called narot, and in the current table is set to 0 by default. Here, we run daverage with a Fourier compensation step ('fc', 1), so that the ouput oa contains the property fweight, that is visualized in dview (Figure 4A). In *Dynamo* the Fourier compensation counts the number of times a rotated particle contributes to a Fourier component. While computing the raw average, each missing wedge is rotated and added into a volume. This volume has the size of a particle in Fourier space, and it is called fweight (a Fourier weight), representing the number of particles that contribute to each Fourier component. Brightness is proportional to the number of particles contributing to a particular Fourier component.

**4.3** Attenuate the effect of the missing wedge in the data set by randomizing the rotational angle narot (a). Average the particles with the new randomized rotational angle (b), so that the Fourier weight map of the average shows a more homogenous distribution of the particles orientations (c) (Figure 4B). Direct space views of the particles along Y show the effect of the missing wedge as a blurred elongation of the image (d) (Figure 4C).

(a) tConsistentRandomized = dynamo_table_ randomize_azimuth(tConsistent);
(b) oaRandomized = daverage(‘particlesData’, 't',tConsistentRandomized,'fc',1);
(c) dview(oaRandomized.fweight);
(d) dmapview({oa.average,oaRandomized. average});

### 5. Rotational alignment STA project

After either performing the section 3 or section 4 (be careful to not mix results from both sections concerning table and average), we create a rotational alignment STA project with appropriate numerical parameters in order to search along the whole sphere for azimuthal angle convergence of particles. After running this project, the average obtained reveals a new structural feature in the crown.

**5.1a** Write the table tr and its corresponding average if particles were oriented as described in section 3 (a) (b).

(a) dwrite(tr, ‘zOriented.tbl’);
(b) dwrite(oz.average, ‘zOriented.em’);

**5.1b** Write the table tConsistentRandomized and its corresponding average if particles were oriented as described in section 4 (a) (b).

(a) dwrite(tConsistentRandomized, ‘zOriented.tbl’);
(b) dwrite(oaRandomized.average, ‘zOriented.em’);

**5.2** Create the rotational alignment STA project called zOriented (a). Introduce the numerical parameters and set the computing environment (see Supplementary Tables S3, S4). Then, check, unfold and run the project as described before (see section 2.3 and Supplementary Table S4). Finally, visualize the resulting average in dmapview (b). Explore results in dmapview (Supplementary Figure S2).

(a) dcp.new(‘zOriented’,'d','particlesData', 'template','zOriented.em','masks', 'default','t','zOriented.tbl');
(b) ddb zOriented:a –m

**Figure 5:**
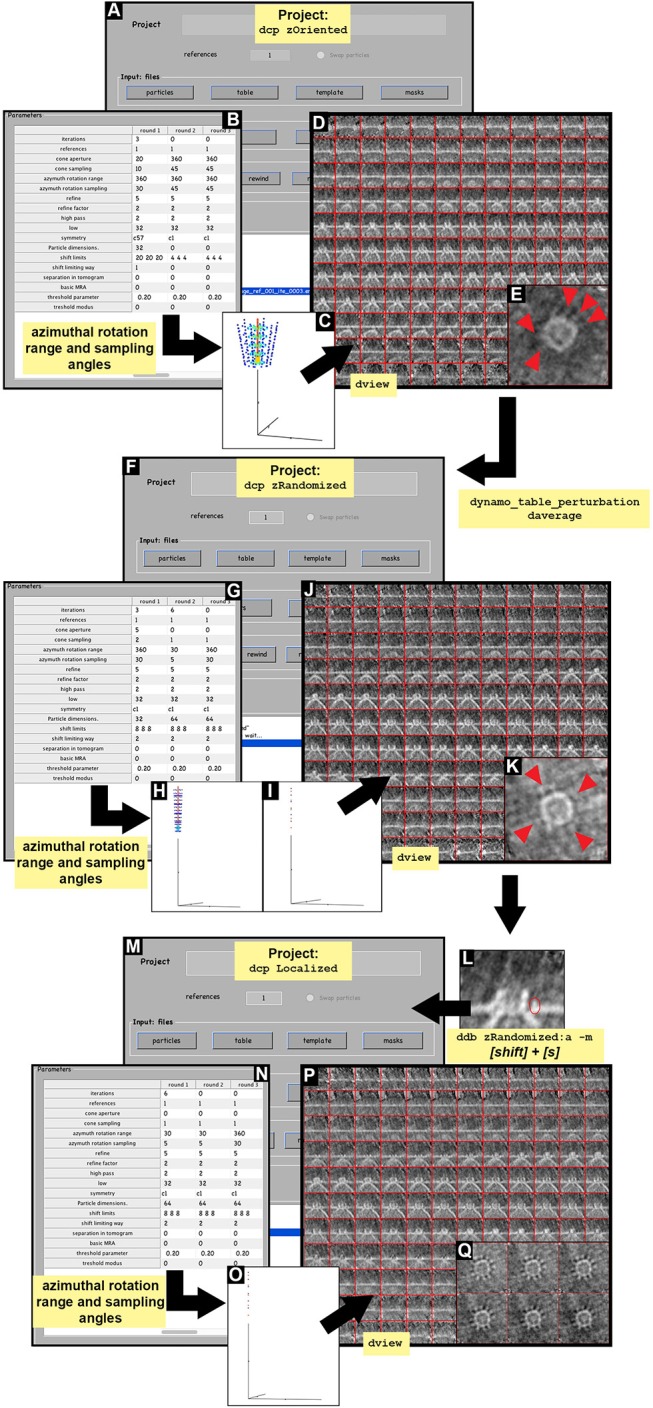
STA projects. Visual pipeline describing the basic steps applied during the STA projects to obtained detailed ultrastructural information. **(A–E)** Basic steps summarizing the design of the STA project zOriented. **(A)** zOriented project GUI where a particle data, table (zOriented.tbl), template (zOriented.em) and mask (default) are used to generate an average. **(B)** Numerical parameters used in **(A)**. **(C)** Graphical representation of the angle search mode defined in **(B)** concerning cone aperture and cone sampling as well as azimuthal rotation range and azimuthal sampling angles. **(D)** Y view of the average obtained as the result of running the STA project zOriented. **(E)** Z view of average slice number 66 in dmapview obtained in **(D)**, red arrowheads indicated ultrastructural details revealed around the crown structure of the FHV docking site. **(F–K)** Basic steps summarizing the design of the STA project zRandomized. **(F)** zRandomized project GUI where particle data is defined. The defined table is resulted from the STA project zOriented after applying the function dynamo_table_perturbation to perform azimuthal randomization of the table (randomized.tbl). The template (randomizedAverage.em) is the average resulting from the STA project zOriented after averaging (daverage) by applying the randomized table (randomized.tbl). The masks are defined as the default. **(G)** Numerical parameter used in **(F)**. **(H,I)** Graphical representation of the angle search mode defined in **(F)** concerning cone aperture and cone sampling as well as azimuthal rotation range and azimuthal sampling angles in round 1 and 2, respectively. **(J)** Y view of the average obtained as the result of running the STA project zRandomized. **(K)** Z view of average slice number 63 in dmapview obtained in **(J)**, red arrowhead indicate ultrastructural details revealed around the crown structure of the FHV docking site. **(M–Q)** Basic steps summarizing the design of the STA project localized. **(M)** Localized project GUI where particle data is defined. The defined table is resulted from the STA project zRandomized. The template is the average resulting from the STA project zRandomized. The alignment mask is defined by manually drawing a mask covering the teeth structure obtained from STA project zRandomized (teethMask.em). The rest of the masks are defined as default. **(L)** Y view of slice number 63 of the average obtained in the STA project zRandomized**(N)** Numerical parameter used in **(M)**. **(O)** Graphical representation of the angle search mode defined in **(F)** concerning cone aperture and cone sampling as well as azimuthal rotation range and azimuthal sampling angles.**(P)** Y view of the average obtained as the result of running the STA project localized. **(Q)** Z view of average slice numbers 58–63 in dmapview obtained in **(P)**.

**INFO:** In the rotational alignment STA project we ask for a symmetrization of c57 to simulate fully rotational symmetry and force any possible symmetry axis in the data along the Z axis: an actual physical symmetry is not assumed. Here, a relevant parameter is the azimuth rotation range, searching for rotations along the full axis in 30° steps (Figures 5A–C and Supplementary Tables S2, S3). Particle alignment and orientation were already completed in previous steps, therefore cone aperture and cone sampling must be restricted to not lose the orientation already achieved.

The results of the project zOriented shown the incipient formation of a crown. The presence of a pointy ring structure surrounding the crown is also hinted at (Figure 5D, red arrowheads). However, the image suffers from the effect of the missing wedge, which manifests itself as striations along one direction (Figures 5D,E).

### 6. Rotational randomization STA project

From the previous section, we have obtained an average manifesting the effect of the missing wedge. In this section we create a template that does not have a preferential orientation by azimuthal randomization of the particles using the table. The randomized template and table are used to feed a new STA project where the resulting average shows clearly the teeth in the crown.

**6.1** Extract the table obtained in zOriented STA project into the workspace variable tns (a) and impose a perturbation of the table by randomly rotating the particles around their normal axis (b) (Figure 5F). Check the average of the particles when applying the randomized table (c) and write the average and the randomized table to feed the next STA project (d).

(a) ddb zOriented:rt -r tns;
(b) tr = dynamo_table_perturbation(tns, 'pshift',0,'paxis',0,'pnarot',360);
(c) oar = daverage('particlesData','t', tr,'fc',1);dview(oar);
(d) dwrite(oar.average,'randomized Average.em');dwrite(tr,'randomized.tbl');

**6.2** Create the STA project called zRandomized (a), check, unfold, run the project as previously described (Supplementary Table S4).

(a) dcp.new('zRandomized','d', 'particlesData','template', 'randomizedAverage.em','masks','default', 't','randomized.tbl');

**INFO:** Numerical parameters are found in Supplementary Table S3 and Figure 5G with a graphical representation of the angle search (Figures 5H,I).

The results of this project show a faintly hinted crown-like structure with a ring of teeth (Figure 5J). The attenuation of the missing wedge shows the isotropic appearance of the teeth, with no stripes along a preferential orientation (Figure 5K, red arrowheads).

### 7. Localized alignment STA project

Features of the structure are revealed after imparting orientation, rotational alignment and randomization of the particles to overcome the missing wedge effect. A crown-like structure surrounded by teeth-like structures is visualized. The teeth-like structures are essential for viral RNA to be exported to the cell cytoplasm, they interact with the OMM opening a tight whole where the crown-like structure is inserted. Hence, the teeth possess high molecular flexibility (Ertel et al., 2017). To improve the signal of the teeth in the final average, we need to focus on this region to run a new STA project where teeth are refined. To do that, we create a mask on the teeth area. The resulting average clearly reveals the location of the teeth allowing crown symmetry calculation.

**7.1** Visualize the average obtained by zRandomized in dmapview (a). Select the Y view of the average in dmapview in a single central slice where the teeth are visible (Figure 5L and Supplementary Figure S2) and press [shift] + [s] on the selected slice. In the new window hand draw a mask on the teeth (Figure 5L). When the mouse is released, the mask is automatically saved in *Dynamo* as a revolution solid by rotating the region drawn about the Z axis, into the file temp_drawn_revolution_mask.em. Change the name of the mask file (b). Create a new project by branching the project zRandomized into the new localized project (c) (Figure 5M). Check, unfold and run the project as previously described depending on the computing environment (see Supplementary Table S4).

(a) ddb zRandomized:a –m
(b) !mv temp_drawn_revolution_mask.em teethMask.em;
(c) dynamo_vpr_branch zRandomized localized -b 1 -noise 0

Note: the character ‘!’ in (b) is MATLAB command to call the operative system.

**INFO:** Numerical parameters are defined in Supplementary Table S3 and Figures 5N,O.

**Figure 6:**
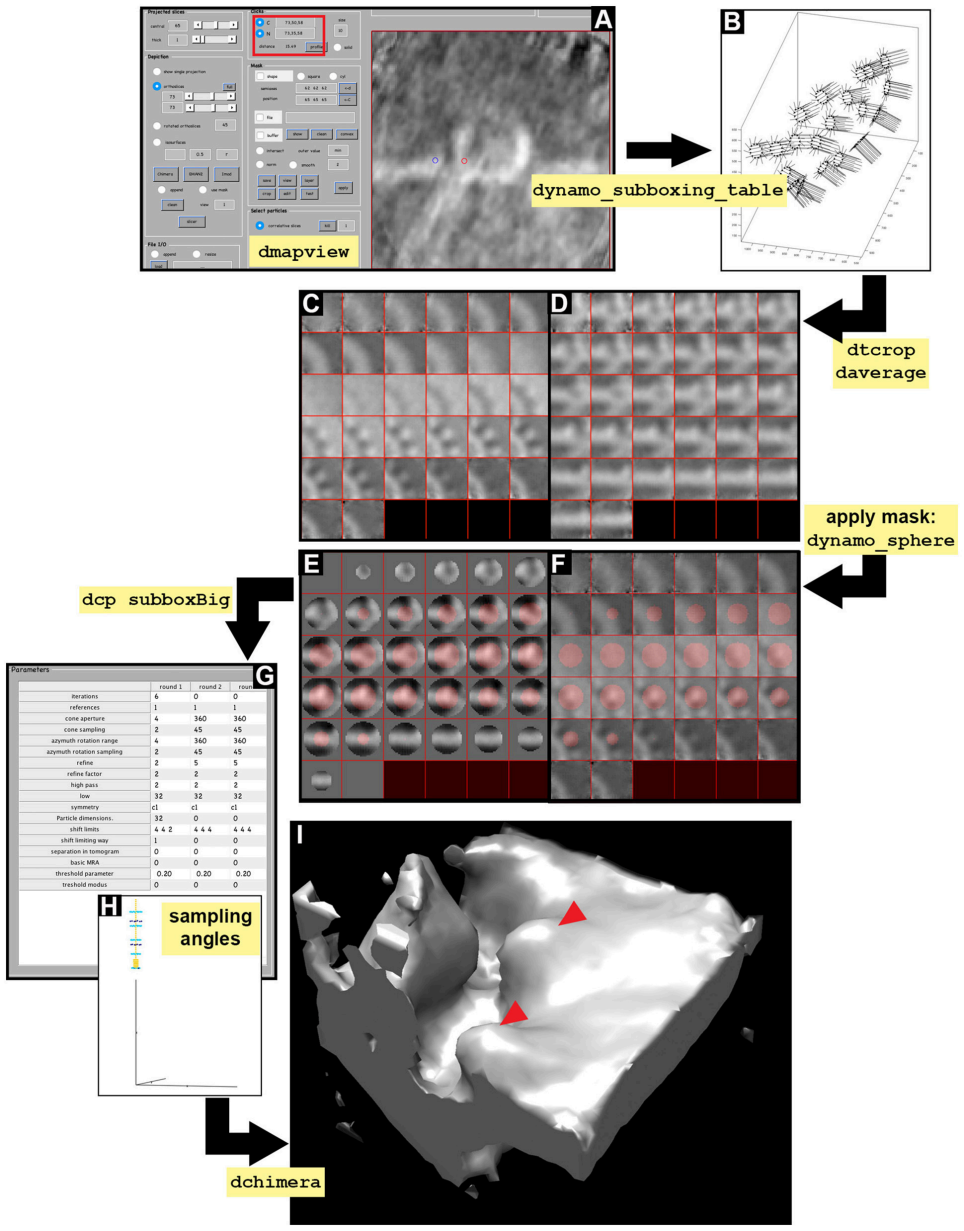
Subboxing STA project. Visual pipeline describing the basic steps taken during the subboxing STA project to obtain detailed ultrastructural information of asymmetrical subunits. **(A)** Y view in dmapview of the average obtained in STA project localized to measure the distance between the crown [red circle, **(C)** button inside the red boxed area] and the tooth (blue circle, N button inside the red boxed area). **(B)** Graphical representation of the particles contained in the sub boxed table (dynamo_subboxing_table). Black points represent the subunits around the crown structure and black lines show their corresponding normal vector. **(C,D)** Z and Y view of the average obtained after performing daverage with the subboxed particles, respectively. **(E,F)** Z and Y view of the average obtained after performing daverage with the subboxed particles and applying the mask dynamo_sphere, respectively. **(G)** Numerical parameter used in the subboxed STA project (dcp subboxBig). **(H)** Graphical representation of the angle search mode defined in **(G)** concerning cone aperture and cone sampling as well as azimuthal rotation range and azimuthal sampling angles. **(I)** Density map of the average result of the execution of the subboxed STA project displayed in chimera (dchimera), red arrowheads indicate the revealed teeth surrounding the crown.

**Figure 7:**
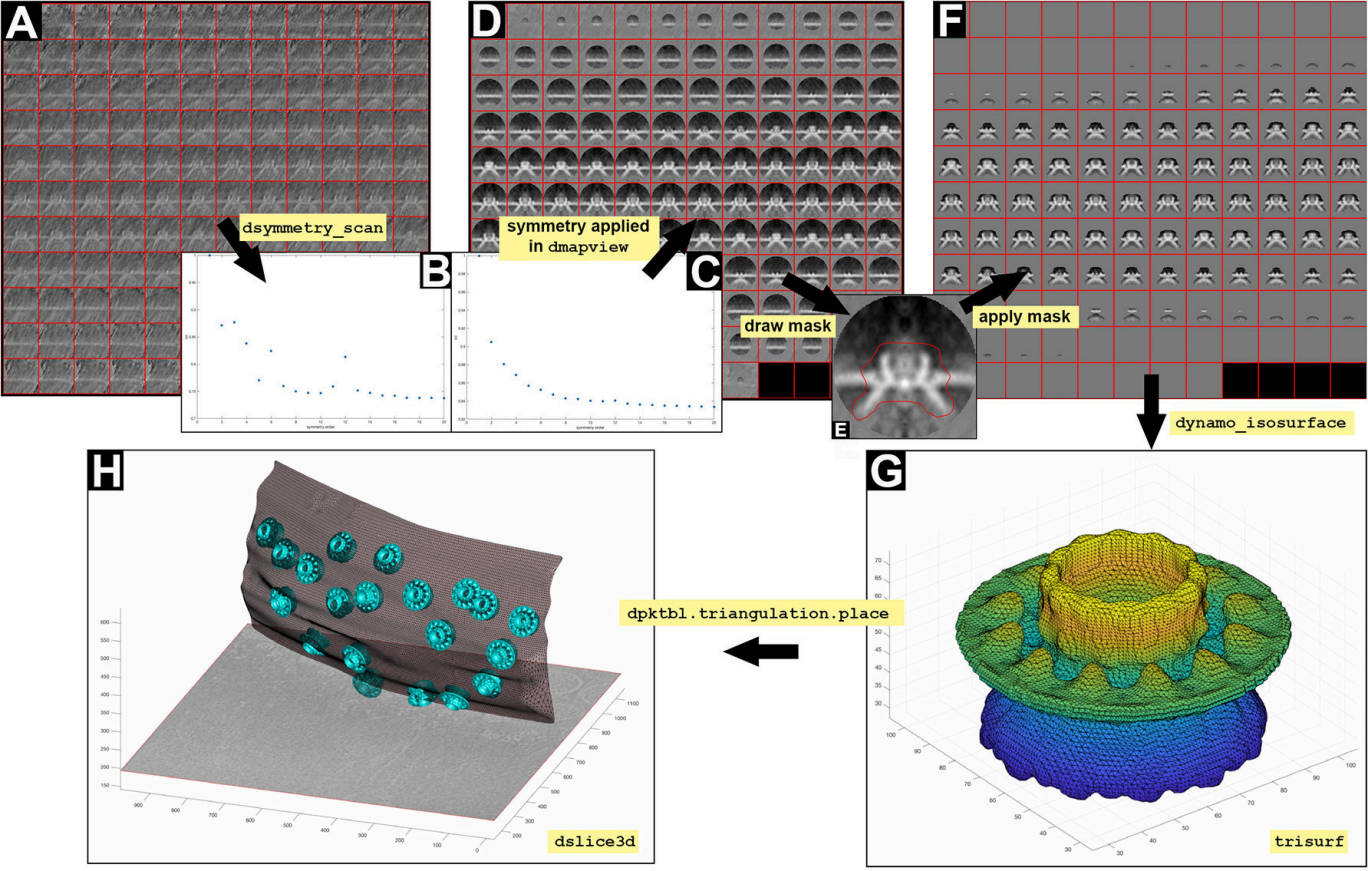
Visualization of 3D scenes from *Dynamo* models and density maps. **(A)** Y view of the average obtained by executing the STA project localized. **(B,C)** Graphical representation of symmetry (dsymmetry_scan) found in **(A)** with and without applying the mask file teethMask.em, respectively. **(D)** Y view of the c12 symmetrized average obtained by executing the STA project localized. **(E)** Y view of the slice number 65 of the average obtained by executing the STA project localized. The mask is defined by manually drawing an area covering the crown structure (red line). **(F)** Y view of the c12 symmetrized average obtained by executing the STA project localized STA after application of the mask defined in **(E)**. **(G)** Graphical reconstruction using trisurf of the structure of the crown as a triangulated surface (dynamo_isosurface). **(H)** 3D displayed (dslice3d) of the crown particles (cyan surface) inserted in the OMM (gray triangulated surface, dpktbl.triangulation.place) in the tomogram.

Note: symmetry per particle can be assessed using the same command described in step 7.2 by changing the input from average volume to particle volume. However, we do not recommend to assess symmetry per particle since it may lead to symmetry artifacts due to lower SNR. In the case that particles suffer from heterogeneity, we recommend to apply classification methods as multireference alignment (MRA), prior to symmetry assessment.

The result of this project provides a clearer insight into the region of interest (Figures 5P,Q). The signal inside the mask is the only signal taken into account, however, the material outside the mask does not become smeared. This corroborates that the particle alignment is robust and not an artifact or imposition. The teeth surrounding the crown are clearly visible and the quality of the average allows a subboxing project to be pursued on the teeth (Figures 5Q, 6). In addition, the localized masking provides a clearer insight into the region of relevance (teeth) and visual inspection of the resulting average after c1-refinement suggest the presence of symmetry. To assess the symmetry of the teeth region we use the following *Dynamo* functionalities:

**7.2** Test the symmetry of the crowns taken as a whole (a) or as the ring of teeth, teethMask.em (b). Symmetry c12 is obtained when applying the teethMask.em mask, meaning that the symmetry of the structure comes from the signal of the ring of teeth forming the crown (Figures 7B,C).

(a) stm = dynamo_symmetry_scan('localized:a' ,'c','order',3:15,'type','pearson', 'nfig',3);
(b) slm = dynamo_symmetry_scan('localized:a' ,'c','order',3:15,'type','pearson', 'mask','teethMask.em', 'nfig',4);

The symmetry in this area appears to be c12. This symmetry can be enforced by symmetrization as described later in section 9 (Figures 7A–C).

### 8. Subboxing

The subboxing technique redefines the area of interest within a previously defined average. For this biological sample, full crowns were the subjects of alignment and averaging, hence, their signal drove the alignment of the particles. Thus far, heterogeneity and flexibility were not considered in this approach. Therefore, potential heterogeneity within particles may decrease the quality of the alignment as the crown is treated as a whole. In the subboxing technique the individual teeth will drive the alignment, so that each particle in the new data set is a subbox (a tooth) extracted from the previous box (the crown). The c12 symmetry observed for the ring of teeth within the crown in the previous section means that the ring is formed by twelve teeth, therefore each crown (box) provides twelve teeth (subbox) for the subboxing STA project (Figure 6A). Here, we show how to locate and extract the teeth (subboxes) from the crown particles (boxes), and run a subboxing STA project to refine the teeth-like structure. As mentioned before, the teeth-like structures possess high biological relevance in the case of FHV due to their man role in viral cytoplasmic infection and propagation.

**8.1** Define the location of a seed subbox (teeth) (a) by activating the anchor points as described in Figure 6A and Supplementary Figure S2. Extract the position of the teeth (b) and the last refined table (c). Define the positions related to t with c12 symmetry along the axis (d). Plot the geometrical relation of teeth (Figure 6B, black dots) and their normal (e) (Figure 6B, black lines).

(a) dmapview localized:a
(b) rSubunitFromCenter = [88,80,53] - [64,64,64];
(c) ddb localized:rt -r t
(d) ts = dynamo_subboxing_table(t, rSubunitFromCenter,'sym','c12');
(e) figure;dtplot(ts,'m','sketch', 'sketch_length',100,'sm',30); view(-151,12);axis equal.

**INFO:** The center of the box (crown) is (64, 64, 64) in pixels and the location of the teeth is (88, 80, 53). We subtract half the sidelength of the full box to reveal the position of the asymmetrical unit expressed in relation to the center of the box.

The new subboxed table ts includes metadata for 12 times as many rows as the original table t, due to the fact that particles (teeth) come from the previous particles (crowns). The system of reference on each particle (teeth) points Z in the direction of its original box (crown). The X and Y orientations of each tooth are symmetrically related to its corresponding box (crown).

**8.2** Create a subboxed data folder that contains the cropped particles (teeth) from the original tomogram suiting the sidelength that we previously obtained in dmapview (32 pixels) (a). Average the new particles (teeth) (b), visualize them (c) and write the average (d).

(a) dtcrop('crop.rec',ts,'subboxData',32);
(b) osb = daverage('subboxData','t',ts, 'fc',1);
(c) dview(osb.average);
(d) dwrite(osb.average,'subboxRaw.em');

**8.3** Create a mask covering the tooth structure (a) and visualize the mask on the average (b, Y view) (c, Z view) (Figures 6E,F). Write the mask into disk (d).

(a) cs = dynamo_sphere(10,32);
(b) figure;dslices(osb.average,'y','ov' ,cs,'ovas','mask');
(c) figure;dslices(osb.average,'ov', cs,'ovas','mask');
(d) dwrite(cs,'maskTooth32.em');

**8.4** Create a subboxed STA project (a). Check, unfold and run the subboxing STA project as described before (Figures 6G–H and Supplementary Tables S2–S4) and check the refined teeth (b). This result can be visualized in Chimera (c) (Figure 6I). In order to path Chimera to *Dynamo* see Supplementary Figure S2.

(a) dcp.new('subboxBig','d','subboxData', 'template','subboxRaw.em','masks', 'default','t','subboxData/crop.tbl', 'show',0).
(b) ddb subboxBig:a:ite = [0,3] –m
(c) ddb subboxBig:a –c

**INFO:** The flag ‘show’, 0 suppresses the dcm GUI from appearing as a window. numerical parameters are defined in Figure 6G and Supplementary Table S2. Another way of entering the numerical parameters into an STA project by the command line is by using dvput as follows:

(1) dvput subboxBig mask maskTooth32.em (the mask is introduced in the project).
(2) dvput subboxBig ite_r1 3 [three iterations are defined for round 1 (ite_r1)]
(3) dvput subboxBig cr_r1 4 (cone aperture in round 1 is defined as 4).
(4) dvput subboxBig cs_r1 2 (cone sampling in round 1 is defined as 2)
(5) dvput subboxBig ir_r1 4 (azimuth rotation range is defined as 4 in round 1)
(6) dvput subboxBig is_r1 2 (azimuth rotation sampling is defined as 2 in round 1)
(7) dvput subboxBig rf_r1 2 (refined is set at 2 for round 1)
(8) dvput subboxBig rff_r1 2 (refine factor is set at 2 for round 1)
(9) dvput subboxBig dim_r1 32 (particle dimensions are defined as 32 pixels in round 1)
(10) dvput subboxBig lim_r1 [4,4,4] (shift limits are set at 4,4,4 pixels in X, Y and Z, respectively)
(11) dvput subboxBig limm_r1 1 (shift limiting way is set at 1 in round 1).

### 9. Creation of 3d scenes

The FHV docking site structure is attached to the OMM and possesses a crown and a ring of 12 teeth that help transition of the viral RNA from the mitochondria into the cell cytoplasm. This structural information is better understood in 3D scenes; therefore, we explain the depiction of 3D scenes using *Dynamo* (Figure 7). We will use the results from section 7 (average and table) to build a 3D figure.

In the case that this section is performed independently of the rest of the protocol described above, see section Equipment set up to download table, template and membrane model, and omit steps 9.3 and 9.4 of this section.

**9.1** Define the tomogram containing the structure as a slice object (a), the source file (b), the center (c), the horizontal and vertical length (d) and the view to be displayed (e). Then, the slice is computed and the data is fetched (f).

(a) s = dpktomo.volume.slices.Slice();
(b) s.source = ‘crop.rec’;
(c) s.center = ‘center’;
(d) s.l = [1000,1000];
(e) s.eulers = ‘z’;
(f) s.fetchData();

**INFO:** The object s reads data from a tomogram file. s.center can be defined as coordinates [x,y,z] (e.g., [256,200,100] (coordinates and dimensions are expressed in pixels).

**9.2** Define a figure (a) and a depicter for the slice object s (b). Then the axis must be considered (c) to create a scene (d). The perspective of the figure can be modified (e).

(a) f = figure; haxis = gca();hold on;
(b) sz = dpktomo.volume.slices. SliceGraphic(s);
(c) sz.axis.h = haxis;
(d) sz.create();
(e) view([-38,60]);

**9.3** Define and write the table containing the metadata information of the particles (crown) that will be placed in the figure (a) (b).

(a) ddb localized:rt –r t;
(b) dwrite(t,'alignmentLocalRefinement. tbl');

**9.4** Extract the average (a) and write it into disk (b) from localized STA project. Then, display the average (c), symmetrize by c12 in dmapview to obtain an improved graphical depiction (see Figure 2 and Supplementary Figure S2) and hand draw a mask on the crown structure as defined in Figures 7D–F and Supplementary Figure S2.

(a) ddb localized:a –r a;
(b) dwrite(a, ‘averagedLocalRefinement. em’);
(c) dmapview averagedLocalRefinement.em;

Note: The hand draw mask is used to assess the symmetry of the masked region.

**9.5** Read the hand draw mask (a) and the template (b), then apply the mask onto the template (c).

(a) maskRevolution = dynamo_read('temp_drawn_revolution_mask .em');
(b) template = dread ('averagedLocalRefinement.em');
(c) final = dsym(template,'c12') ^*^maskRevolution;

**INFO:** The notation .^*^ indicates the pixelwise multiplication of two volumes. So that we can obtain a final volume for 3D representation were only the region of interest is included.

**9.6** Write the final template (a) and visualize it (b).

(a) dwrite(final,'placedTemplate.em');
(b) dview placedTemplate.em;

**9.7** Define a new figure (a) and create the triangulation for the template (b) as well as its corresponding surface (c).

(a) f2 = figure();
(b) dt = dynamo_isosurface(final,'isolevel' ,0.79,'real_isolevel',true,'-show', false);
(c) trisurf(dt.tr);axis equal;

**INFO:** The object dt contains the triangulation parameters.

The result is a triangulation of a representative isosurface of the average. Now, we proceed to insert copies of the average where the particles are inserted (crowns, Figure 7G) in the OMM.

**9.8** Extract the table from the localized STA project (a) and create a triangulation object that merges the result of copying, relocating and rotating the isosurface of one template on all the positions identified in the table (b).

(a) t = dread('alignmentLocalRefinement. tbl');
(b) tAll = dpktbl.triangulation.place(t, dt.tr,'rc',64.5);

**INFO:** The number 64.5 indicates the position of the rotation center for each box (crown). Remember that the particles were cropped with a sidelength of 128 pixels.

**9.9** Use the depiction figure (a) to display all triangulated averages corresponding to the positions of the particles described in the table (b). Adjust figure color (c) and style (d). Set some display features, such as name of figure (e), functionality of mouse cursors to zoom, drag and rotate the viewpoint (f), shading (g) and angles (h). Define a colormap for the tomogram slice and the averages (i), and set again the color of the averages again (j).

(a) figure(f);
(b) hTriangulation = trisurf(tAll); shg;
(c) hTriangulation.FaceColor = 'c';
(d) hTriangulation.LineStyle = 'none'; shg;
(e) axis equal; f.Name = 'Simple trisurf scene';
(f) mbgraph.cursors.setMouse3d([]); shg;
(g) shading(haxis,'faceted');
(h) l = lightangle(-45,30); shg);
(i) ocm = dynamo_colormap();shg;
(j) hTriangulation.FaceColor = 'c';

**INFO:** Coexistence of two or more colormaps needs to be simulated in MATLAB by using the *Dynamo* function dynamo_colormap. The command shg; is used to bring the figure to the front.

**9.10** Refresh the figure (a) and move the depicted slice to satisfy visualization (b).

(a) s.refresher.dataUpdatesAfter GeometryChanges();sz.surface. autoUpdate();
(b) s.shift([0,0,100]]);

**INFO:** By doing step (a) any change in thickness, center, and axis, among others, will be automatically refreshed in the figure, e.g. shift the slice upwards.

**9.11** Read the OMM model (a) and extract its triangulation into the figure (b) (Figure 7H).

(a) m = dread('membraneModel.omd');
(b) hMem = trisurf(m.mesh.tr);

**INFO:** Note that ‘membraneModel.omd’ refers to the path of the file containing the surface model. The model path may change depending on the location of the file and the name given. If section 3.2 was performed to get the surface model, this model should be still contained in the workspace variable m, therefore, step (b) in section 9.11 can be directly applied.

## Conclusion

STA is a unique method used to elucidate the structure of *in situ* macromolecular complexes. It is increasingly used in the field of electron tomography now that improvements in microscope hardware facilitate the visualization of vitrified cells and eukaryotic lamellas (Briggs, 2013; Villa et al., 2013; Wan and Briggs, 2016). In tomography, particles of interest are imaged distributed in 3D space along with other components of the cellular context. Thus, tomography projects present several biological scenarios that differ from one another on the level of particle distribution. For example, while some particles of interest might be embedded in a membrane, other might be distributed in the cell cytoplasm or associated with cellular organelles. This requires a broad range of approaches to extract ultrastructural information, and might require the development of complex tailored approaches on a case-by-case basis.

Here, we describe a detailed protocol customized for a particular geometrical case (isolated particles irregularly distributed in a direction imparted by a membrane). In this data set, FHV macrocomplexes are imaged at close-to-native state within an intact mitochondrion. Visualized FHV particles are structurally arranged as they would be in nature, inserted in the OMM. Thus, the OMM is presented as a common feature among the FHV particles, making an appropriate candidate to drive particle alignment not only due to convergence but also to signal.

In other biological scenarios, e.g., proteins decorating cytoskeletal protein filaments, the filament will act as the FHV OMM driving the particle alignment. In the case of reconstituted membrane proteins in liposomes (forming proteoliposomes), the liposomal membrane drives particle alignment. However, in the case of purified or cytoplasmic proteins and viral capsid proteins, the protein signal drives particle alignment. In this scenario, an alignment mask would be appropriate to exclude noise signal originated by the buffer, the cytoplasm or neighboring cytoplasmic proteins. Such biological samples, e.g., ribosomes (Khoshouei et al., 2017), HIV capsid proteins (Schur et al., 2016), are suitable for high-resolution STA due to their sampling abundancy and overall homogeneity. Furthermore, the missing wedge effect on such particles is not so acute since they are randomly distributed, and therefore, not suffering from preferred orientation.

In contrast, membrane or filament attached macrocomplexes, e.g., FHV and microtubule-bound dynein-dynactin complexes (Grotjahn et al., 2018), are aligned based on such conspicuous features. In this cases, particle alignment should be feasible in few iterations on a STA project, however, protein signal convergence may be challenging in cases where membrane/filament signal is much stronger than the protein signal or particles suffered from heterogeneity respect to their interaction with the membrane/filament feature. Such challenges may be solved by the appropriate use of masks during STA projects as well as investing higher number of iterations for refinement.

Ultrastructural studies concerning cell biology are often interested in answering a relevant biological question rather than aiming for the highest resolution possible. In the case of FHV, the main biological question is focused on the ultrastructural characterization of the viral crown; how viral spherules interact with the OMM to transfer their RNA genome to be replicated and therefore, propagate within the cell cytoplasm? This biological question was answered by the visualization of ‘teeth’ at the viral crown by STA. Teeth are shown to disrupt the OMM by creating a channel for RNA transference into cytosol. However, other structural biology projects involving *Dynamo* for STA pursue high-resolution cryo-ET. Such projects are focused on estimating final average resolution as key to assess particle convergence and refinement. *Dynamo* estimates the resolution through the customarily called *golden standard method*. An alignment project ran in adaptive bandpass modus inside *Dynamo* will estimate the resolution of the attained average through the calculation of the Fourier Shell Correlation (FSC) (Harauz and van Heel, 1986) of two half averages computed independently at each iteration. Particle convergence is measured in a regular STA project by computing the FSC between the last and previous computed average.

In such STA projects thousands of particles are involved, therefore, extensive computational resources are convenient to improve effectiveness, as e.g., the use of GPU accelerators. These are especially suitable for subtomogram averaging tasks, where the computational burden lays on the comparison of many rotated versions of the same volume (the template) against the same data particle. This is a close to ideal scenario for the use of GPUs, whose architecture explicitly devises optimal performance for intensive processing of a given data piece. Speed up factors in the approximate range of 200x have been reported in Castaño-Díez (2017).

For the case described in this protocol GPU devices are not required due to low number of particles and iterations during the STA project involved in this stepwise procedure.

To conclude, this protocol describes the operative logic of the *Dynamo* software package, showcasing the repertory of tools available for geometric manipulations. The protocol offers information about *Dynamo* commands that are routinely used during STA, so that the user can become familiar with the *Dynamo* environment. The procedure is divided into several modules that can also be taken individually to solve punctual issues that arise when tomographic data are analyzed. Even though the data set is specific, the problems presented are not unique, but common to many STA projects, e.g., automatic assignment of asymmetry (subboxing) and inherent loss of Fourier information in presence of preferential views (missing wedge). Thus, many of the tools explained in this protocol can be applied to a broad range of STA scenarios for tomographic data, and promise to inspire the development of new STA strategies while the *Dynamo* software package is being used.

## Author contributions

PN performed the numerical experiments and wrote the manuscript with contributions from all authors. HS provided infrastructure and scientific advice. DC-D conceived the image analysis workflow.

### Conflict of interest statement

The authors declare that the research was conducted in the absence of any commercial or financial relationships that could be construed as a potential conflict of interest.

## Abbreviations

2D: two-dimensional
3D: three-dimensional
Cryo-ET: cryo-electron tomography
ET: transmission electron tomography
FHV: flock house nodavirus
GUI: graphical user interface
MPI: message passing interface
OMM: mitochondrial outer membrane
RAM: random-access memory
SNR: signal-to-noise ratio
STA: subtomogram averaging.
