# Supplementary material for: Protocols for Subtomogram Averaging of Membrane Proteins in the Dynamo Software Package
Source: Front Mol Biosci. 2018 Sep 4;5:82. doi: 10.3389/fmolb.2018.00082 (PMC6131572; doi:10.3389/fmolb.2018.00082)
Supplement: Supplementary file 1 [file Data_Sheet_1.DOCX]

**Supplementary Material**

**Protocols for subtomogram averaging of membrane macrocomplexes in the *Dynamo* software package**

**Paula P. Navarro, Henning Stahlberg, Daniel Castaño-Díez***

*** Correspondence:** Daniel Castaño-Díez: daniel.castano@unibas.ch

**Supplementary Figures and Tables**

**Table S1: *Dynamo* basic commands**

| **Section** | **Command** | **Function** | **Modifiers** | **Modifiers effect** |
| --- | --- | --- | --- | --- |
| *Tomographic data management* | dynamo_catalogue_manager or dcm | Opens *Dynamo* *catalogue* GUI | -create  -at  -l tomogram | Create new *catalogue*  Add data to the *catalogue*  List items of a given category, in this case tomograms |
|  | dtmslice(); | Opens *Dynamo dtmslice* GUI | -c  -prebinned | *Catalogue* name  Bin factor |
|  | dfile(); | Read file providing file type and size information |  |  |
|  | dtmshow(); | Inspect image file or volume | -otf (on the fly) | Indicates to not preload the full tomogram, but to access in disk the individual slices needed. |
|  | dynamo_catalogue_bin(); | Bin tomogram (bin factor needs to be indicated) | -zchunck | Maximum number of Z slices that are kept simultaneously in the memory during the binning process. |
|  | dcmodels(); | Gives the model information of a *catalogue* | -nc  -ws  -i l | Name of model contains  Defines a workspace variable for the indicated *model*  List of model indices |
|  | dread(); | Read file |  |  |
|  | m.grepTable(); | Extract a variable from an object (m), in this case the table of a catalogued model called ‘m’. |  |  |
|  | dtinfo(‘*name of the table file’*); | Gives a summary of the information coded in a table |  |  |
|  | dtcrop(); | Crops particles out of a tomogram |  |  |
|  | daverage(); | Average particles applying a table. Often used to create a template. |  |  |
| *Project design: particle alignment* | dwrite(); | Write files in disk |  |  |
|  | dcp.new(); | Creates a new STA project |  |  |
|  | dvcheck(); | Indicates a possible error in the STA project |  |  |
|  | dvunfold(); | Creates a script to run the STA project |  |  |
|  | ddb(); | *Dynamo* data base | -v  -m  -c | Send output of ddb query to the depiction GUI called dview.  Send output of ddb query to the depiction GUI called dmapview  Send output of ddb query to Chimera |
|  | ddbrowse(); | *Dynamo* data browse | -d  -t | Load data  Load table |
| *Aligning the axis of symmetry: strategy A: without geometric information* | dpktomo.examples.motiveTypes.MembraneWithRod(); | Creates a geometrical object with the shape of a membrane with a rod. | sidelength  rodRadius  rodShift  rodHeight  getMask();  viewMask(); | Define dimension  Define radius of the rod  Define position of the rod  Define height of the rod  Harness the rod as a mask  Display mask in dview |
|  | dalign(); | Align particle data against a mask |  |  |
|  | dynamo_table_rigid(); | Modifies a table according to the geometrical information of a rigid body |  |  |
|  | dtplot | Plots data |  |  |
| *Aligning the axis of symmetry: strategy with geometric information* | dpktbl.triangulation.fillTable(); | Impart an orientation to the point of a given table |  |  |
|  | dynamo_table_flip_normals(); | Flip the normal of a table according to the given center point |  |  |
|  | dchimera(); | Opens chimera from *Dynamo*. Often used to export data directly from *Dynamo* to Chimera | -path | Add chimera path from your local system |
|  | m.ezplot(); | Depict model (in this example: m) following and indicated option, i.e., surface |  |  |
| *Management of the missing wedge* | dynamo_table_randomize_azimuth(); | Randomize the rotational angle of the particles |  |  |
|  | dmapview(); | Open GUI to explore and compare averages |  |  |
| *Project for rotational randomization* | dynamo_table_perturbation(); | Impose a perturbation in a given table, keeping the rest of parameters constant but randomly rotates the particles around their axis of symmetry (narot). |  |  |
| *Project for localized alignment* | dynamo_vpr_branch(); | Branch a given project into a new one keeping all settings from the given project. |  |  |
| *Subboxing project* | dynamo_subboxing_table(); | Creates a subboxed table out of a boxed table |  |  |
|  | dynamo_sphere(); | Creates a sphere that can be used as a mask |  |  |
|  | dvput(); | Enter numerical parameters values |  |  |
|  | dvhelp(); | List the names of the numerical parameters used in a STA project |  |  |
| *3D scenes* | dslice3d(); | Create a orthogonal slice of a given tomogram to the indicated direction (x, y or z) that cuts a defined point. |  |  |
|  | dpktomo.volume.slices.Slice(); | Creates slices from a given tomogram along with geometrical parameters | source  center  l  fetchData  eulers | Defines tomogram  Defines center of the displayed tomogram  Defines length of the displayed tomogram  Compute defined slice of a given tomogram and parameters  Imparts a rotation |
|  | dpktomo.volume.slices.SliceGraphic(); | Depict a defined object (i.e., slice from a tomogram) | axis  create | Create an axis based on the data depicted  Create the slice in the available axis |
|  | shg(); | Brings figure to front |  |  |
|  | dsym(); | Symmetrize a given average |  |  |
|  | dynamo_isosurface(); | Creates an isosurface based on a given average |  |  |
|  | trisurf(); | Display triangulation of a given object |  |  |
|  | dpktbl.triangulation.place(); | Create a triangulation object |  |  |
|  | dynamo_colormap(); | Display selected colors on a triangulated object |  |  |

|  |  |  |  |  |
| --- | --- | --- | --- | --- |

**Table S2: dcp GUI Numerical Parameters**

| **Name** | **Command line name** | **Definition** | **Tips** |
| --- | --- | --- | --- |
| **Iterations** | ite | Number of iterations to be performed in a given round (i.e., during a given round, several iterations are performed with the same parameters). | Enter zero to skip a round. |
| **References** | nref | Number of references used during the iteration. Each reference will generate its own average and its own cross correlation matrix (ccmatrix). References are completely independent from each other: there is no interaction between them and they act as separate computation channels. | For multireference alignment see online documentation. |
| **Cone aperture** | cr | The first two Euler angles are used to define the orientation of the vertical axis of the protein. First Euler angle (tdrot) rotates the template around its z axis. Second Euler angle (tilt) rotates the template around its x axis. *Dynamo* scans for this axis inside a cone: The 'cone_range' parameter defines the angular aperture of this cone. | 360 degrees is thus the value for a global scan.  *To skip the part of the angular search that looks for orientations, you have to set:*  *1)* 'cone range' *to zero, and*  2) 'cone_sampling' to 1. |
| **Cone sampling** | cs | *Dynamo* scans the best orientation of the axis of the reference inside a cone. The cone sampling parameter expresses the discretization inside this cone. The sampling is given in degrees, and corresponds to a representative angular distance between two neighboring orientations inside the cone. |  |
| **Azymuth rotation range** | inplane_range or ir | The third Euler angle (narot) defines rotations about the new axis of the reference. 360 degrees is the value for a global scan. | To skip the part of the angular search that looks for azimuthal rotations and\or orientations, set: |
| **Azymuth rotation sampling** | inplane_sampling or is | Defines the interval of sampling for the azymutal range (degrees). It is associated with the narot angle (New Axis ROTation).  the axis of the template is rotated to a new orientation (defined by tdrot and tilt).  The rotated template rotates again on its new axis (an ‘inplane’ rotation).  It defines the angular interval (in degrees) between two of these inplane rotations. | 'inplane range' to zero, and  'inplane_sampling' to 1. |
| **Refine** | refine or rf | Number of refinement levels to compute in each single particle. Multilevel alignment compares a set of rotations of the reference against the data, selecting the best orientation and searching again with a finer sampling. The sampling in the refine search is half the sampling used in the original. The range of the refinement search encompasses all orientation that neighbor the best orientation found in the original search |  |
| **Refine factor** | refine_factor or rff | Controls the size of the angular neighborhood during the local refinement of the angular grid. |  |
| **High pass** | high | High pass threshold or filter (in Fourier pixels) | If your template is smaller than the data, the bandpass parameters will be rescaled |
| **Low** | low | Los pass threshold or filter (in Fourier pixels) | If your template is smaller than the data, the bandpass parameters will be rescaled |
| **Symmetry** | sym | Symmetry is applied at the beginning of the round to the input reference. | First letter indicate the type of symmetry:  ‘c’: rotation symmetry around z  ‘h’: helical symmetry around z  ‘ico’: icosahedral symmetry  ‘cbo’: cuboctahedral symmetry. |
| **Particle dimensions** | dim | Resamples the particles to the specified dimensions (pixels). Template and mask will be resampled accordingly. This value represent the full cube sidelength. | This is a useful parameter to bin the particles when running an averaging project. The dimension of the cropped particles is 128 pixels in the original tomogram, those can be binned by a factor of 2 for the project to run faster for this first alignment project, so the particle dimension are set to 32 pixels. |
| **Shift limits** | area_search or lim | Restricts the search area to an ellipsoid centered and oriented in the last found position. The three parameters are the semiaxes of the ellipsoid. If a single parameter is introduced, the ellipsoid collapses into a sphere. | If no restriction should be imposed, put a zero on the ‘area_search_modus’ project parameter.  The position 15 of the table keeps a record on how far apart was the real maximum of the cross correlation value from the restricted maximum. |
| **Shift limiting way** | area_search_modus or limm | States interpretation of parameter ‘area_search’.   - 0: no limitations - 1: limits are understood from the center of the particle cube - 2: limits are understood from the previous estimation on the particle position. The origin of the shifts changes at each iteration. - 3: limits are understood form the estimation provided for the first iteration of the round. The origin of the shifts will change at each round. - 4: limits are understood from the estimation provided for the first iteration of the project. The origin of the shifts is thus defined for the full project, and stays static during the full computation. | Option 3 and 4 are useful to avoid particle gradually shifting away from the initially user-entered locations. |
| **Separation in tomogram** | separation_in_tomogram | Minimum separation allowed between the position of two particles (pixels). If the value is a positive number *Dynamo* checks relative positons of all the particle in each tomogram separately. Whenever two particles are close together than ‘separation_in_tomogram’, only the particle with the higher correlation stays. The other is deleted from the table. |  |
| **Basic MRA** | mra | Activate the Multi Reference Alignment (MRA). It swaps particle from reference to reference according to CC score. |  |
| **Threshold parameter** | Threshold or thr | Threshold value to select which particle are averaged in vies of their cross correlation value (CC). |  |
| **Threshold modus** | threshold_modus or thrm | Interpretation of the threshold parameter.   - 0: no thresholding policy - 1: threshold parameter is an absolute threshold. Only particle with CC above this value are selected. - 2: effective threshold = mean(CC)* threshold_parameter. - 3: effective threshold = mean(CC)+std(CC)*Threshold_parameter - 4: threshold_ parameter is the total number of particle (ordered by CC). - 5: Threshold ranges between 0 and 1 and sets the fraction of particles. |  |

**Table S3: *Numerical Parameters* values for each project of the protocol.** First column defines the name of each parameter. The rest of the columns concerns one project each and defines the value taken by the parameters. Note that all projects complete one round except project zRandomized that completes two rounds.

| **Parameter** | first **project** | zOriented **project** | zRandomized **project** | | Localized **project** | subboxBig **project** |
| --- | --- | --- | --- | --- | --- | --- |
| **Round** | 1 | 1 | 1 | 2 | 1 | 1 |
| **Iterations** | 4 | 3 | 6 | 1 | 6 | 6 |
| **References** | 1 | 1 | 1 | 1 | 1 | 1 |
| **Cone aperture** | 360 | 20 | 5 | 0 | 0 | 4 |
| **Cone sampling** | 30 | 10 | 2 | 1 | 1 | 2 |
| **Azymuth rotation range** | 0 | 360 | 360 | 30 | 30 | 4 |
| **Azymuth rotation sampling** | 1 | 30 | 30 | 5 | 5 | 2 |
| **Refine** | 5 | 5 | 5 | 5 | 5 | 2 |
| **Refine factor** | 2 | 2 | 2 | 2 | 2 | 2 |
| **High pass** | 2 | 2 | 2 | 2 | 2 | 2 |
| **Low** | 32 | 32 | 32 | 32 | 32 | 32 |
| **Symmetry** | c1 | c57 | c1 | c1 | c1 | c1 |
| **Particle dimensions** | 32 | 32 | 32 | 64 | 64 | 32 |
| **Shift limits** | 20 20 20 | 20 20 20 | 8 8 8 | 8 8 8 | 8 8 8 | 4 4 2 |
| **Shift limiting way** | 1 | 1 | 2 | 2 | 2 | 1 |
| **Separation in tomogram** | 0 | 0 | 0 | 0 | 0 | 0 |
| **Basic MRA** | 0 | 0 | 0 | 0 | 0 | 0 |
| **Threshold parameter** | 0.20 | 0.20 | 0.20 | 0.20 | 0.20 | 0.20 |
| **Threshold modus** | 0 | 0 | 0 | 0 | 0 | 0 |

**Table S4: Computational environment in *Dynamo*.**

| **Environment** | **Platform** | **Definition** | **Run a *Dynamo* STA project** |
| --- | --- | --- | --- |
| **MATLAB** | Windows, Linux, Mac | Run project on one single core within a MATLAB environment. | dcp GUI (press bottom RUN) or type ‘*name of the project’* in MATLAB |
| **MATLAB (parfor modus)** | Windows, Linux, Mac | Start parallel pool with available MATLAB workers specified by the user. | dcp GUI (press bottom RUN) or type ‘*name of the project’* in MATLAB |
| **Standalone** | Windows, Linux, Mac | Run project on the cores specified by the user within a standalone environment. | ./’*name of the project.exe’** |
| **GPU (under MATLAB)** | Linux, Mac | Run project on GPU (number of GPUs specified by the user) within a MATLAB environment. | dcp GUI (press bottom RUN) or type ‘name of the project’ in MATLAB |
| **GPU (standalone)** | Linux, Mac | Run project on GPU (number of GPUs specified by the user) within a standalone environment. | ./’*name of the project.exe’** |
| **Cluster MPI** | Linux | Run project on a cluster of CPUs controlled by an MPI parallelization protocol. | Queue submission |
| **Cluster MPI with GPUs** | Linux | Run project on a cluster of GPUs controlled by an MPI parallelization protocol. | Queue submission |

*In a terminal where *Dynamo* is activated but the *Dynamo* console is not running (‘Dynamo>’ does not appear in the terminal).

Note: To check number of available cores in Linux: go to the dcp GUI press on the bottom computing environment and go to *Tools> Check number of available cores (Linux)*. In a MATLAB environment to check number of available cores go to the dcp GUI press on the bottom computing environment and go to *Parallel Toolbox> Check cores available for MATLAB Parallel Toolbox*.


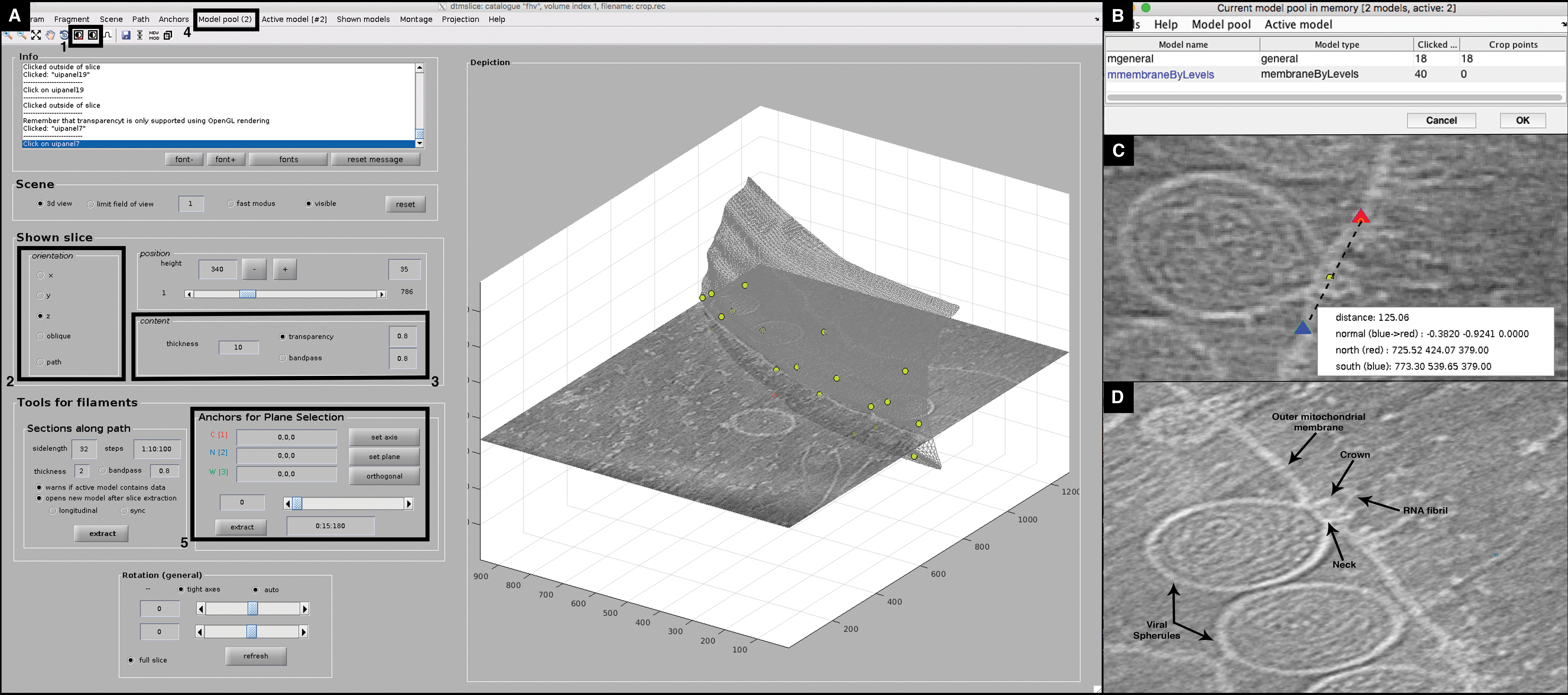


**Supplementary Figure S1. dtmslice basic functionalities.** (A) dtmslice command opens a *Dynamo* GUI for visualization and annotation of tomograms. Different parameters are displayed: image contrast (box 1), plane of view (box 2), projection thickness, transparency and bandpass (box 3). One of the main functions of dtmslice is particle picking. Picking particles in *Dynamo* throughout dtmslice involves creating models *(*box 4*, Model pool>create new model in pool)* of specific geometries resembling the distribution pattern of the biological structure of interest within the tomogram. (B) The manually created models reside in memory (*Model pool>Open current model pool as a table*) until they are saved in disk, into the *catalogue*. To save a model into the *catalogue* click on: *Active model>save active model into catalogue (disk)* or to save all current models in pool: *Model pool> Save all models into catalogue*. Create a general model (*Model pool>Create new model in pool (choose type)>general*) containing the picked particles concerning the FHV docking site, the neck. The currently active model is visible in the dtmslice scene, and particles are picked, and thereby added to the model, by pressing [c] when the tip of your mouse is positioned on the particle of interest. A direct map between user clicks and putative positions of particles is visualized in the dtmslice depiction window (A and C, green dots). Press the key ‘delete’ in the keyboard to delete last clicked particles. (C) Right click on a specific particle and a menu with the metadata information of the point will be shown as well as the option delete, among others. Importantly, bear in mind that the particle-picking step is essential for STA. Selected points positioned in the center of the structure to consider will be used as mark to extract the subvolumes from the tomographic data, and subset them in a data folder with the proper format. To do this, we need to analyze the sidelength of the particles within the tomogram (in pixels) by pressing the keys [1] and [2], which define two anchor points that appear as blue and red rhombohedral points in dtmslice, respectively. Both rhombohedral points are linked by a dashed line. Information concerning coordinates, length and normal direction can be accessed by clicking on the dashed line, and displayed in the ‘Anchors for Plane Section’ in the GUI (box 5). (D) Biological features that can be seen composing and surrounding the particles (FHV docking site).


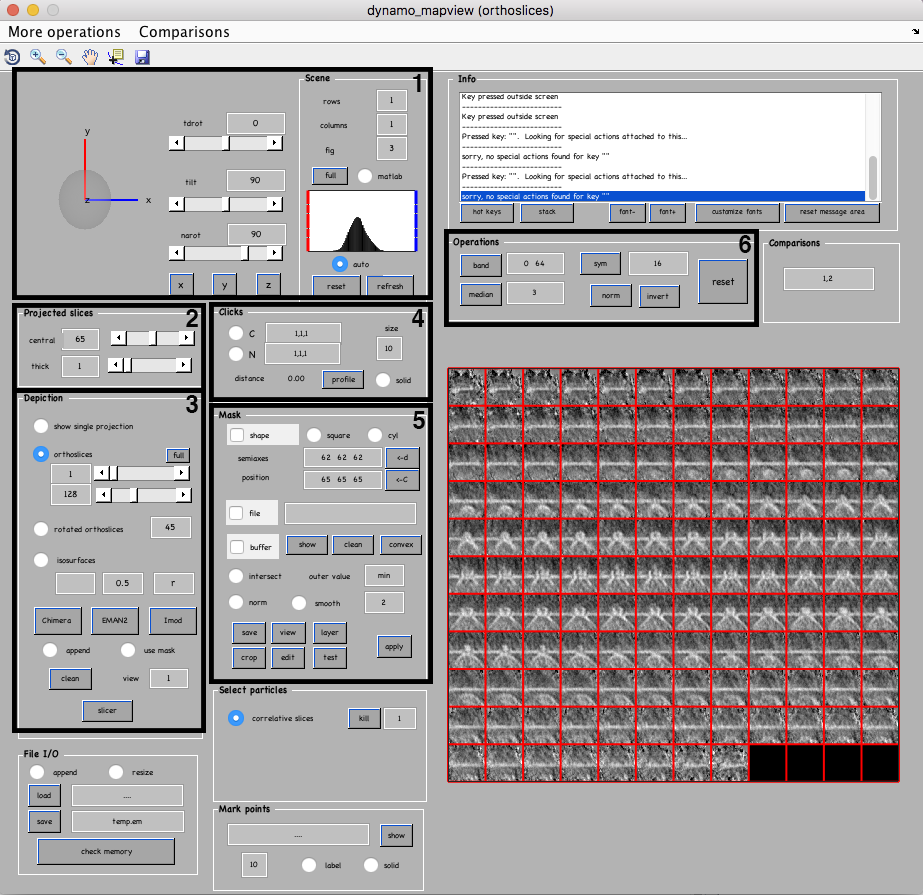


**Supplementary Figure S2.** dmapview **GUI.** dmapview is a *Dynamo* GUI that helps to explore averages obtained from STA projects. Several averages can be loaded in order to compare the changes that occur when applying different parameters and geometrical transformations are applied. For example, in Figure 4C improvements concerning the missing wedge effect when randomizing the table is shown by dmapview({oa.average,oaRandomized.average});. Depiction of the evolution of the average from the first template to iteration number can be visualized in dmapview through: ddb zOriented:a:ite=[0,3] –m. Iterations 0 and 3 will be visualized in dmapview. To visualize all iterations use the command: ddb zOriented:a:ite=*. In box 1 the view X, Y, Z as well as the rotation angles (tdrot, tilt, narot) can be changed. A sphere with X, Y, Z axis helps to visualize the orientation displayed. Furthermore, display options can be modified as well as the contrast (left clicking on the contrast graph changes the red dashed line to the clicked area and right clicking changes the blue dashed line to the clicked area). Box 2 defines the central slice and the thickness of the average displayed. Box 3 helps to visualize specific orthoslices; to see specific orthoslices of the average drag the arrow buttons or to see all orthoslices of the average press the bottom ‘full’. Rotation and isosurfaces can be applied and the density map can also be sent to software like Chimera*, EMAN2 and IMOD when pathed to *Dynamo*. Box 4 presents the markers C (center, left clicking) and N (north, right clicking), that can be activated and clicked to measure distances and position sub-structures within the average. In Box 5 a mask can be designed with several shapes, saved and applied onto the average. When pressing the bottom 'layer' a new dview window opens visualizing the average with the mask applied to it in red. Box 6 describes different operations that can be applied to the average, such as bandpass filtering, normalization and symmetry among others.

**Dynamo* includes a link to send maps into Chimera UCSF, EMAN2 and IMOD. To make these tools functional *Dynamo* needs to know the location of Chimera UCSF, EMAN2 or IMOD. Taking Chimera UCSF as an example, in a MATLAB environment type dynamo_chimera -path myChimeraPath In the standalone version, set the path to Chimera UCSF before initiating the *Dynamo* session in your shell, for instance: export PATH=$PATH:myChimeraPath or in a running *Dynamo* session type: mbsys.os.addPath('*myAbsoluteChimeraPath*');
