# Supplementary figures and images for: Protocols for Subtomogram Averaging of Membrane Proteins in the Dynamo Software Package
Source: Front Mol Biosci. 2018 Sep 4;5:82. doi: 10.3389/fmolb.2018.00082 (PMC6131572; doi:10.3389/fmolb.2018.00082)

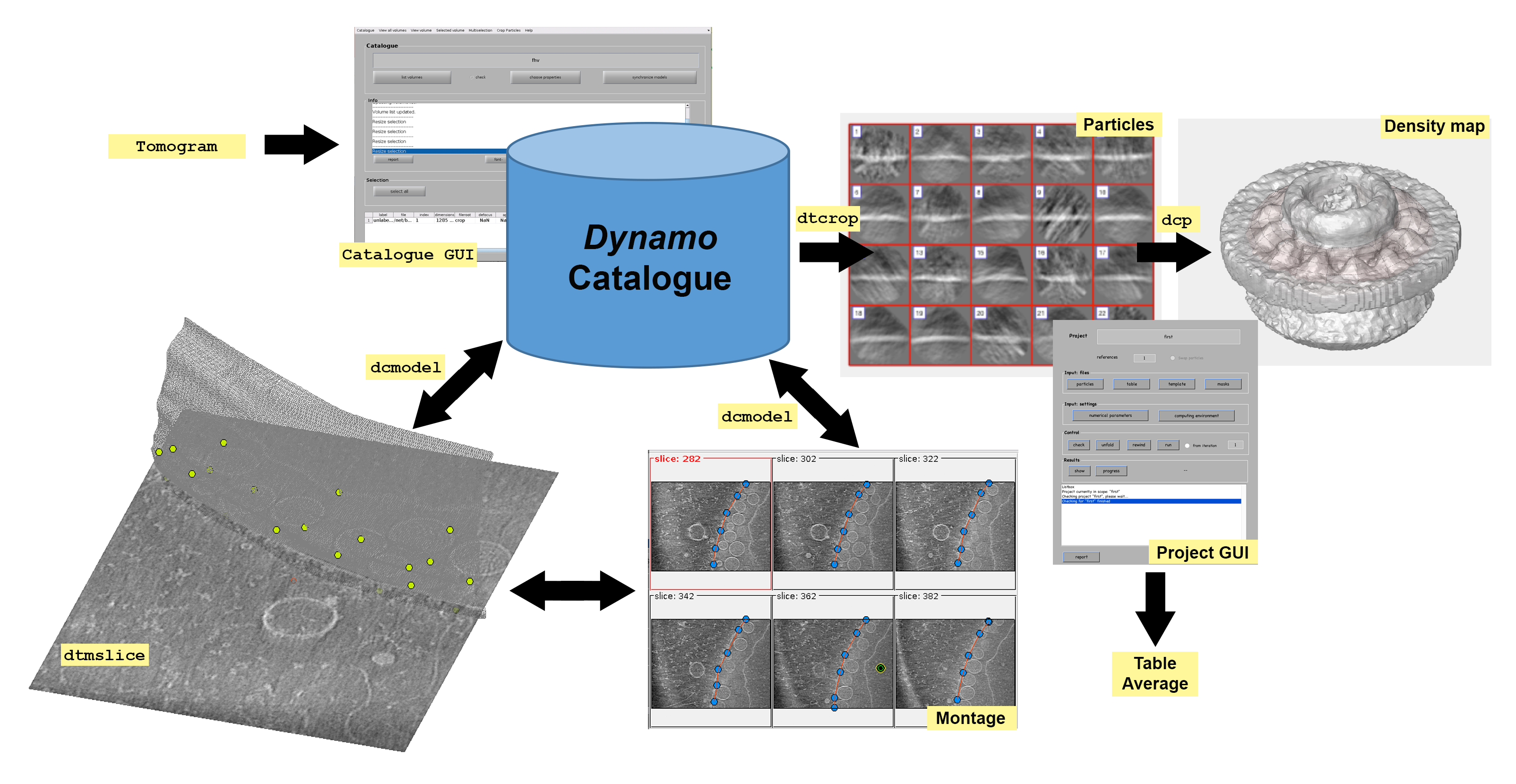

Supplement: Supplementary file 2 [file Image_1.TIF]
